# Quantized Information in Spectral Cyberspace

**DOI:** 10.3390/e25030419

**Published:** 2023-02-26

**Authors:** Milton A. Garcés

**Affiliations:** 1 Infrasound Laboratory, University of Hawaii, Kailua-Kona, HI 96740, USA; milton@isla.hawaii.edu; 2 RedVox, Inc., Kailua-Kona, HI 96740, USA

**Keywords:** Gabor atoms, logons, quantum wavelet, Fourier transform, wavelet transform, Stockwell transform, wavelet entropy, spectral entropy, cyberspectral information, spectral cyberspace, relative entropy, signal processing, Tonga, Lamb wave, acoustics, infrasound, acoustic-gravity wave

## Abstract

The constant-Q Gabor atom is developed for spectral power, information, and uncertainty quantification from time–frequency representations. Stable multiresolution spectral entropy algorithms are constructed with continuous wavelet and Stockwell transforms. The recommended processing and scaling method will depend on the signature of interest, the desired information, and the acceptable levels of uncertainty of signal and noise features. Selected Lamb wave signatures and information spectra from the 2022 Tonga eruption are presented as representative case studies. Resilient transformations from physical to information metrics are provided for sensor-agnostic signal processing, pattern recognition, and machine learning applications.

## 1. Introduction

The transformation of digital time-series into spectral information can enhance signal detection, exploration, and feature extraction for machine learning (ML) applications. This paper expands and generalizes a standardized [1], quantized computational framework [2] within the context and nomenclature of Gabor [3] and Cohen [4,5,6,7,8,9], and integrates the ensuing concepts and methods with wavelet [10,11,12,13,14,15,16] and Stockwell [17,18,19,20,21] transforms. The resulting spectral power metrics are then aligned with Shannon information and entropy metrics [9,22,23,24,25,26,27,28,29,30,31,32,33,34,35,36] to facilitate the fusion of multi-modal data streams from heterogeneous sensor systems [37].

Section 2, Methods, concentrates on the implementation of the fast Fourier transform (FFT) [38], the computationally efficient implementation of the discrete Fourier transform. Relative entropy metrics are developed and evaluated for time–frequency representations obtained from the continuous wavelet transform (CWT) [10,11,12,13,14,15,16,21], the Stockwell transform (STX) [17,18,19,20,21], and the short-time Fourier transform (STFT) [38,39,40], using readily available open-source FFT [41,42,43] algorithms. The concept of an information and entropy signal-to-noise ratio e.g., [2] is further standardized relative to a uniform distribution, with possible extension to any other reference distribution.

Section 3, Algorithm Evaluation and Results, expands the spectral information methods and applies them to Tonga Lamb wave signatures representative of energetic broadband transients. Selected spectral information and entropy representations illustrate some of the different types of information that can be embedded in a signal’s energy signature. However, this is not a geophysics paper; it is an invitation to change perspective from physical to entropy metrics for pattern recognition and ML applications, where normalization, linear, and nonlinear transformations are inevitable, and stable, reproducible, and explainable methods are preferable.

Section 4, Discussion, summarizes the impact of the transformation from signals in physical space to multiresolution spectral entropy representations. Proposals are presented for possible future studies of broadband transients such as Lamb waves and other types of signatures that may be well matched to the methods presented herein.

### 1.1. Spectral Cyberspace

The preferred staging for the governing relations in nondimensionalized computational (cyber) spaces requires reconciliation and standardization of cyber and physical time and frequency scales for digital sensor systems with different dynamic ranges and diverse sample intervals. Most mathematical solutions are implicitly solved in such cyberspaces; in general, one may rescale only at the beginning and at the end of computations. Let $t_{p},\;{\tilde{f}}_{p}$ be the physical time and physical frequency that would be observed in the real world. Time–frequency measurements in the digital cyber-physical signal processing domain are assumed to be sampled at a nominal time interval $\Delta \tau_{s\;}$ with a corresponding sample rate $\;{\tilde{f}}_{s\;}=1/\Delta \tau_{s\;}$. Unless otherwise stated, all equations refer to nondimensionalized (cyber) time, defined as

(1) $$t={\tilde{f}}_{s\;}t_{p},$$

with cyber frequency $\tilde{f}$ and angular frequency ω

(2) $$\tilde{f}=\frac{{\tilde{f}}_{p}}{{\tilde{f}}_{s\;}};\;\omega =2\pi \tilde{f}=2\pi \frac{{\tilde{f}}_{p}}{{\tilde{f}}_{s\;}}.$$

Temporal discretization is set by the sample interval, whereas the frequency may be discretized in many ways. The resulting digital time–frequency grids will be referred to as *spectral cyberspace* or *cyberspectral space*, and may be conceived as the canvas containing spectral information in cyber time–frequency space. The CWT and the STX are exceptionally flexible, which can lead to unreasonable frequency oversampling or undersampling. The STFT inherits the frequency resolution given by the FFT window duration. The temporal resolution of the STFT is generally given by the percent overlap, or hop length, of the sliding FFT window. In contrast, both the CWT and STX can retain the same temporal resolution as the input signal and have more malleable frequency specifications. Although the multiresolution CWT and STX methods work exceptionally well for logarithmically-spaced frequency scales, one may also specify linearly spaced frequencies. The STFT can use a variety of tapering windows [39]. The Gabor CWT and STX use a Gaussian envelope, which has the unique property of minimizing the time–frequency uncertainty of their spectral representations.

### 1.2. Fundamentals of the Gabor Logon

The compromise between time and frequency energy (and entropy) concentration is succinctly expressed by the Heisenberg–Gabor uncertainty principle, where the temporal and angular frequency variance of the marginals [5,9] satisfy

(3) $$\sigma_{t_{n}}^{2}\sigma_{\omega_{n}}^{2}\geq \frac{1}{4}\;.$$

The canonical Gabor–Morlet wavelet, also referred to as the Gabor logon, atom, or grain, satisfies the minimum uncertainty requirement with the general canonical form

(4) $$\Psi_{n}\left(t\right)=G_{n}\left(t\right)\;e^{j\omega_{n}t}$$

where

(5) Gnt=1πσn214exp−12tσn2

with standard deviation $\sigma_{n}=\sqrt{2}\sigma_{t_{n}}$ (e.g., [2] and Appendix A).

An alternate scaling of the Gaussian envelope has gained substantial popularity in practical applications, particularly for ML with multiresolution spectral distributions e.g., [13,14,15],

(6) gnt=14πσn214Gnt

(7) $$g_{n}^{2}\left(t\right)=\frac{1}{2\pi \sigma_{n}^{2}}exp\left\{-{\left[\frac{t}{\sigma_{n}}\right]}^{2}\right\}$$

(8) $$\psi_{n}\left(t\right)=g_{n}\left(t\right)\;e^{j\omega_{n}t}\;,$$

with the advantage of a spectrum with a peak of unity for each band when using the asymmetric Fourier transform pair (Appendix B)

(9) $${\hat{g}}_{n}\left(\omega \right)=\int_{-\infty }^{\infty }g_{n}\left(t\right)\;e^{-j\omega t}dt,$$

(10) $${\hat{g}}_{n}\left(\omega \right)=exp\left\{-\frac{1}{2}{\left[\omega \sigma_{n}\right]}^{2}\right\}\;.$$

This is the favored Gaussian envelope for the STX [17,18,19,20,21] and for advanced ML applications of the CWT [13,14,15].

For a given reference time scale $\tau_{0}$ and a logarithmic scale base *G > 1*, a quantized center time scale $\tau_{n}$ and frequency $\;{\tilde{f}}_{n}$ can be defined as

(11) $$\frac{\tau_{n}}{\tau_{0}}=G^{\frac{n}{N}},{\tilde{f}}_{n}=\frac{1}{\tau_{n}}\;,$$

where *n* is the band number and *N* is the band order [1,2]. Appendix A demonstrates that the critical number of cycles $M_{N}$ in a Gaussian window with standard deviation $\sigma_{n}$ scales with the angular frequency $\omega_{n}=2\pi /\tau_{n}$ as

(12) $$M_{N}=\frac{3\pi }{4}N$$

(13) $$\sigma_{n}=\frac{M_{N}}{\omega_{n}}=\frac{3}{8}N\;\tau_{n}$$

with time support per wavelet

(14) $$T_{n}=M_{N}\tau_{n}=\frac{3\pi }{4}N\;\tau_{n}$$

(15) $$T_{n}=2\pi \;\sigma_{n}=\sqrt{8}\pi \;\sigma_{t_{n}}\sim 9\;\sigma_{t_{n}}\;.$$

The temporal support window $T_{n}$ represents the minimum number of digital points required to process a signal using a quantized constant-Q Gabor atom [2] with a center time scale $\tau_{n}$ of order N. The window $T_{n}$ should be a power of two when using FFT. Albeit succinct, Equation (12) is one of the most important expressions in this paper as its implementation reduces spectral leakage without introducing power gaps between frequency bands (Appendix A). A Gaussian taper window with adequate support performs comparatively well [39], with low side lobes; it has the added benefit of mathematical ease of use. With order and standardized frequencies as organizing principles, the quantized Gabor atom permits the construction of a complete, transportable, and reproducible spectral cyberspace with minimal uncertainty. A sparse representation of the selected signal features and metrics in terms of a reduced number of quantum atoms [2] will inherit their minimal spectral uncertainty.

The rest of this paper uses quantum atoms as basis functions. The reader is invited to verify that the energy of these types of functions only depends on the Gaussian amplitude envelope and scaling, and their integrals over adequate support (Equation (12)) are stable.

### 1.3. Gabor Logon Information and Entropy

Williams et al. [9] follow the nomenclature of Cohen [5] to quantify the Gabor logon information. Please refer to Appendix B and Appendix C for additional details. The instantaneous power per wavelet, or intensity per unit time, is only dependent on the Gaussian envelope.

(16) $$p\left(t\right)={\left|\Psi_{n}\left(t\right)\right|}^{2}={\left|G_{n}\left(t\right)\right|}^{2}=G_{n}^{2}\left(t\right)$$

where the instantaneous power, referred to as the time marginal, is normalized to unity

(17) $$\int_{-\infty }^{\infty }p\left(t\right)dt=1\;.$$

Note the authors in [9] use the canonical form in Equation (4). The symmetric Fourier transform pair (Appendix B)

(18) $${\hat{G}}_{n}\left(\omega \right)=\frac{1}{\sqrt{2\pi }}\int_{-\infty }^{\infty }G_{n}\left(t\right)\;e^{-j\omega t}dt$$

yields the Fourier transform of the Gaussian envelope. The spectral energy density, also referred to as the frequency marginal, is

(19) $$\hat{P}\left(\omega \right)={\left|{\hat{G}}_{n}\left(\omega \right)\right|}^{2}$$

where the total energy in the frequency domain is also normalized

(20) $$\int_{-\infty }^{\infty }\hat{P}\left(\omega \right)d\omega =1\;.$$

The Shannon information [22,23] $I_{t}$ is expressed in bits and defined as the binary logarithm of the probability p, which can be represented by the marginal

(21) $$I_{t}=log_{2}\;\left(1/p\right)=-log_{2}\;p\;.$$

The Shannon entropy $H_{t}$ is also in bits and is the expected value over the distribution, with a familiar discrete representation (with time replaced by *m*)

(22) $$I_{t}\left(m\right)=-log_{2}\;p_{m}.$$

(23) $$H_{t}=-\sum_{m}p_{m}\;log_{2}\;p_{m}=\sum_{m}h_{t}\left(m\right)\;.$$

The information and entropy representations in the frequency domain (with frequency replaced by *k*) are

(24) $$I_{\omega }\left(k\right)=-log_{2}\;{\hat{P}}_{k}$$

(25) $$H_{\omega }=-\sum_{k}{\hat{P}}_{k}\;log_{2}\;{\hat{P}}_{k}=\sum_{k}h_{\omega }\left(k\right)\;.$$

As discussed in Appendix C, the Wigner-Ville [44,45] distribution provides no additional information about the logon beyond that provided by the marginals

(26) $$H_{t\omega }-\left(H_{t}+H_{\omega }\right)=0$$

where, like its uncertainty, the joint time–frequency entropy of each logon is constant

(27) $$H_{t\omega }=log_{2}\left[\pi e\right]\;\sim \;3\;.$$

These relations serve as foundational building blocks for spectral entropy representations. A valuable property of entropy is that, like energy, it is additive when conditioned properly. Although most mathematical papers scale the wavelet to construct the entropy, other signal- or application-specific scaling strategies can be applied on the computed distribution. Due to the inherently nonlinear nature of information and entropy metrics, scaling can suppress or enhance different spectral features imbedded in the raw data.

## 2. Methods

The mathematical optimization of signal representations in spectral cyberspaces has been a subject of considerable study [1,2,3,4,5,6,7,8,9,10,11,12,13,14,15,16,17,18,19,20,21]. This work develops standardized spectral information and entropy metrics that can enhance feature-rich broadband transient signatures.

The signal variance, or energy, of a properly preconditioned signal $x\left(m\right)$ with Fourier transform $\hat{x}\left(\omega_{k}\right)=F\left\{x\left(m\right)\right\}$, can be readily computed. The Fourier transform is approximated by the FFT

(28) $$\hat{x}\left(\omega_{k}\right)=\sum_{m=0}^{M-1}x\left(m\right)e^{-j\omega_{k}m}=F\left\{x\left(m\right)\right\}$$

(29) $$x\left(m\right)=\frac{1}{M}\sum_{k=0}^{M-1}\hat{x}\left(\omega_{k}\right)e^{j\omega_{k}m}=F^{-1}\left\{\hat{x}\left(\omega_{k}\right)\right\}\;,$$

(30) $$\omega_{k}=2\pi \frac{k}{M},\;k=0,\;1,\;2,\ldots \left(M-1\right)\;.$$

The sum of the instantaneous power in the time domain is a reliable measure of the total energy in the time domain

(31) $${\left\|x\right\|}^{2}=\sum_{m=0}^{M-1}{\left|x\left(m\right)\right|}^{2}\;.$$

In the frequency domain, the scaled sum of the Fourier coefficients over all frequencies $\hat{x}\left(m\right)$ is

(32) $${\left\|\hat{x}\right\|}^{2}=\frac{2\pi }{M}\sum_{k=0}^{M-1}{\left|\hat{x}\left(k\right)\right|}^{2}$$

and should numerically meet Parseval’s identity within computational errors

(33) $$\sum_{m=0}^{M-1}{\left|x\left(m\right)\right|}^{2}=\frac{1}{M}\sum_{k=0}^{M-1}{\left|\hat{x}\left(k\right)\right|}^{2}=\frac{2}{M}\sum_{k=0}^{M/2-1}{\left|{\hat{x}}_{R}\left(k\right)\right|}^{2}$$

where ${\hat{x}}_{R}\left(k\right)$ is the real FFT (RFFT), which traditionally only computes the positive frequencies and has half the number of points. The variance in the signal is a quick and useful checksum metric to compare against the FFT coefficients.

### 2.1. Short-Time Fourier Transform (STFT) with a Gabor Atom (GTX)

The localized STFT implements the FFT over sliding tapered windows. The Gabor transform (GTX) uses a fixed-duration Gaussian window as the taper, and can be expressed as [6,20]

(34) $$S_{gtx}\left(\tau ,\omega ,T\right)=\int_{-\infty }^{\infty }x\left(t\right)g_{T}^{*}\left(t-\tau \right)\;e^{-j\omega t}dt$$

where τ is the hop time, which has a constant striding interval in most practical implementations. We can express this as a convolution ⊛, with

(35) $$z\left(t,\omega \right)=x\left(t\right)\;e^{-j\omega t}$$

(36) $$S_{gtx}\left(\tau ,\omega ,T\right)=\int_{-\infty }^{\infty }z\left(t,\omega \right)g_{T}^{*}\left(t-\tau \right)dt=z⊛g_{T}^{*}\;.$$

In discrete cyber time (Appendix B), this can be expressed as

(37) $$S_{gtx}\left(\tau_{m},\omega_{k},T_{p}\right)=\sum_{m^{\prime }=0}^{M-1}x\left(m^{\prime }\right)g_{T}^{*}\left(m^{\prime }-m\right)e^{-j\omega_{k}\frac{m}{M}}$$

(38) $$\omega_{k}=2\pi \frac{k}{M},k=0,\;1,\;2,\ldots M-1$$

(39) $$S_{gtx}\left(\tau_{m},\omega_{k}\right)=F^{-1}\left\{\hat{z}\;{\hat{g}}_{T}^{*}\right\}\;.$$

Cohen and Loughlin [6] evaluate and discuss the marginals of the STFT in more detail. The Welch periodogram e.g., [43] can be readily computed from taking the average of the STFT power over time.

### 2.2. Continuous Wavelet Transform (CWT) with Gabor Atoms

The CWT with Gabor atoms is well known [10,11,12] and can be expressed as,

(40) $$S_{cwt}\left(\tau ,\omega ,n\right)=\int_{-\infty }^{\infty }x\left(t\right)g_{n}^{*}\left(t-\tau \right)\;e^{-j\omega_{n}\left(t-\tau \right)}dt=x⊛\psi_{n}^{*}\;.$$

In the discrete digital time, this can be expressed as

(41) $$S_{cwt}\left(\tau_{m},\omega_{n}\right)=\sum_{m^{\prime }=0}^{M-1}x\left(m^{\prime }\right)\psi_{n}^{*}\left(m^{\prime }-m\right)=x⊛\psi_{n}^{*}\left[m\right]$$

which can also be evaluated as a product in the Fourier domain (Appendix B),

(42) $$S_{cwt}\left(\tau_{m},\omega_{n}\right)=F^{-1}\left\{\hat{x}\;{\hat{\psi }}_{n}^{*}\right\}\;.$$

### 2.3. The Stockwell Transform (STX) with Gabor Atoms

The STX [17,20] is a hybrid between the localized Fourier and wavelet transform, with generalized form

(43) $$S_{stx}\left(\tau ,\omega ,n\right)=\int_{-\infty }^{\infty }x\left(t\right)g_{n}^{*}\left(t-\tau \right)\;e^{-j\omega t}dt\;.$$

As with the STFT, we can express this as a convolution, with

(44) $$S_{stx}\left(\tau ,\omega ,n\right)=\int_{-\infty }^{\infty }z\left(t,\omega \right)g_{n}^{*}\left(t-\tau \right)dt=z⊛g_{n}^{*}\;.$$

The differences and similarities between the three algorithms are exposed, with

(45) $$S_{stx}\left(\tau_{m},\omega_{n}\right)=F^{-1}\left\{\hat{z}\;{\hat{g}}_{n}^{*}\right\}$$

(46) $$S_{gtx}\left(\tau_{m},\omega_{k}\right)=F^{-1}\left\{\hat{z}\;{\hat{g}}_{T}^{*}\right\}$$

(47) $$S_{cwt}\left(\tau_{m},\omega_{n}\right)=F^{-1}\left\{\hat{x}\;{\hat{\psi }}_{n}^{*}\right\}$$

where

(48) $$z\left(t,\omega \right)=x\left(t\right)\;e^{-j\omega t}$$

(49) $$\psi_{n}^{*}=g_{n}^{*}\left(t\right)\;e^{-j\omega_{n}t}\;.$$

Although time–frequency representations return complex coefficients for all frequencies between the negative and positive Nyquist frequencies, for a real signal input only the positive frequencies are sufficient and displayed. The FFT of a real-valued array (RFFT) is generally used for real time series analyses and returned by time–frequency representation (TFR) algorithms. The power in a TFR of a real input function can be estimated from

(50) $$P_{tfr}\left(\tau ,\omega \right)=2{\left|S_{tfr}\left(\tau ,\omega \right)\right|}^{2},\omega \in \left[0,\pi \right]$$

and, when computed with the same scaling, can be fairly compared. Standardizing spectral cyberspaces for comparing heterogeneous sensors with different sample rates, dynamic ranges, sensitivities, and passbands can be challenging without a clear strategy and framework. The next section provides some practical guidelines.

### 2.4. Spectral Staging

Advance preparation and signature tuning are key to satisfactory spectral analyses. A fundamental best practice is to remove constant biases (DC offsets) or linear amplitude trends in the time series within the passband of interest. This step often reveals undesirable characters (Section 2.4.2) and other glitches in the cyber-physical matrix that will require resolution. These two initial preconditioning and quality control stages will help stabilize spectral cyberspaces. The following subsections review additional important considerations and recommended practices.

#### 2.4.1. Sample Interval and Nyquist

The sample interval is the average time between adjacent digital samples. This interval is usually set by the analog to digital converter (ADC) or a data polling algorithm e.g., [37]. In high-fidelity audio and geophysical ADCs, the sample interval is stable and tuned to an internal oscillator that returns even sample intervals with small standard deviations. Nonetheless, these systems can still produce data with gaps (i.e., during power or communication outages) and time drifts which must be addressed with care. Other systems may use a data polling method, where they only record a sample when there is a change from the previous value exceeding a given threshold. The polling method generally leads to uneven sample intervals with substantial standard deviations.

Regardless of the sampling method, a mean sample rate can be estimated from the inverse of the mean sample interval. Twice the sample interval defines the Nyquist interval, and one half the sampling frequency is the corresponding Nyquist frequency e.g., [46]. In a properly configured sampling system, there is little to no energy at the Nyquist frequency to reduce aliasing, the folding of energy about Nyquist. If data are severely aliased down to the bandpass of interest, it may not be useable for spectral analyses. Therefore, the theoretical upper frequency band edge of any digital system is the Nyquist frequency. In practice, the upper band edge is at the half power (−3 dB) cutoff frequency of the low-pass anti-aliasing (AA) filter, which in hi-fidelity ADCs is ~80% of Nyquist. Spectral coefficients above the AA cutoff will be heavily distorted and attenuated. Upsampling does not increase the useable upper band edge.

#### 2.4.2. Input Signal Duration

The number of points in an input signal should be a power of two for the efficient evaluation of spectra with FFTs e.g., [38]. The original record is zero padded if it does not meet this specification. Signal preconditioning is recommended before invoking the FFT. Although most FFT algorithms will zero pad e.g., [43], the results may not be as expected if the signal is aliased, has a substantial DC offset, or contains not-a-number (nan) or other undefined values. It is recommended that FFT computations, including filtering, are performed with the highest floating point precision that balances the required fidelity with available computational resources.

The spectral resolution of the FFT is the sample rate over the number of points in the original or zero-padded signal (which should be a power of two). This sets the lower edge of the passband. Although the FFT returns the zero frequency, a properly conditioned, unaliased signal with zero mean will have no substantial energy at the zero frequency or the Nyquist frequency. The record duration and the sample interval define the maximal time–frequency canvas for the TFR.

#### 2.4.3. Signal Center Frequency and Bandwidth

Ideally, signals of interest will have a maximal concentration of energy at a predictable center frequency and passband. It is desirable to design the TFR around this center frequency and to match the envelope around the energy concentration. This is where the quality (*Q*) factor [1,2], or order (*N*), of the wavelet is useful; both increase with the number of oscillations per wavelet window and, from the uncertainty principle, narrow the wavelet bandwidth (Appendix A). Sustained oscillations, such as musical notes, are well captured by tight high-order bands such as 12th and 24th octave bands. Short duration broadband blasts may be better captured by broader 3rd or 6th octave bands. Mallat ([12], Section 4.2.2, p. 99–100) provides additional useful metrics and examples to quantify bandwidth and spectral spreading.

#### 2.4.4. Averaging Frequency

The record duration may greatly exceed the duration of a signal, or various signals of interest may occur multiple times within a record. The averaging frequency, which should be lower than the signal center frequency and at the lower end of its bandwidth, but larger than the inverse of the (possibly padded) signal duration, provides a lower frequency specification to the spectral analysis and produces an averaging wavelet filter with a nominal duration of 2^J^ points. The averaging wavelet sets the lower frequency band edge. The concept of the averaging filter is an integral part of scattering wavelets [13,14,15]. The averaging wavelet duration can be larger than the original signal duration, and zero padding with tapers can be used to extend the signal (within reason) and efficiently interpolate in the frequency domain. An additional specification is implicit in STFTs, where one must select a sliding, overlapping window that is substantially shorter than the total record duration, and often shorter than the averaging window. Fortunately, most STFT algorithms e.g., [43] have independent input parameters for the sliding window and the FFT window to facilitate zero padding.

#### 2.4.5. Ordered Dyadic Framework

As outlined in Appendix A, the band order *N* is used as the key organizing principle. The band number of the standardized frequency closest to the physical averaging frequency ${\tilde{f}}_{av}$ is

(51) $$n_{av}=\mathrm{round}\left[\frac{N}{log_{2}\left(G\right)}\left\{log_{2}\left({\tilde{f}}_{av}/{\tilde{f}}_{p0}\right)\right\}\right]$$

where ${\tilde{f}}_{p0}$ is the reference physical frequency, and G is the base

(52) $$G=2\;\mathrm{or}\;10^{\frac{3}{10}}\;\mathrm{recommended}.$$

The dyadic number of points $M_{J}=2^{J}$ needed to support the averaging atom is

(53) $$log_{2}M_{J}=\;\mathrm{ceil}\left[log_{2}\left(M_{N}\tau_{0}\;G^{\frac{n_{av}}{N}}\right)\right]$$

where $\tau_{0}$ and $M_{N}$ are defined in Equations (11) and (12). Using fractional octave bands of order *N* with reference period $\tau_{p0}$, or reference frequency ${\tilde{f}}_{p0}=1/\tau_{p0}$ in physical units (e.g., seconds or Hertz, respectively), a record duration of $2^{J}$ points can be represented by quantized Gabor atoms [2] defined by an ordered number of increasing scales with band number *n*, where

(54) $$n_{min}\leq n\leq n_{max}$$

(55) $$n_{min}=\mathrm{ceil}\left[\frac{N}{log_{2}\left(G\right)}\left\{1-log_{2}\left(\;{\tilde{f}}_{s\;}/{\tilde{f}}_{p0}\right)\right\}\right]$$

(56) $$n_{max}=\mathrm{floor}\left[\frac{N}{log_{2}\left(G\right)}\left\{log_{2}M_{J}-log_{2}M_{N}-log_{2}\left(\;\;{\tilde{f}}_{s\;}/{\tilde{f}}_{p0}\right)\right\}\right]\;.$$

The cyber period $\tau_{n}$, frequency ${\tilde{f}}_{n}$, and atom scale per band $\sigma_{n}$ are

(57) $$\tau_{n}={\tilde{f}}_{s\;}\tau_{pn}={\tilde{f}}_{s\;}\tau_{p0}\;G^{\frac{n}{N}}=\tau_{0}\;G^{\frac{n}{N}}$$

(58) $${\tilde{f}}_{n}=\frac{{\tilde{f}}_{\mathrm{pn}}}{{\tilde{f}}_{s}}=\frac{{\tilde{f}}_{p0}}{{\tilde{f}}_{s}}\;G^{-\frac{n}{N}}={\tilde{f}}_{0}\;G^{-\frac{n}{N}}$$

(59) $$\sigma_{n}=\frac{3\pi }{4}\;\frac{N}{\omega_{n}}=\frac{3}{8}\;\frac{N}{{\tilde{f}}_{n}}=\frac{3}{8}N\;\tau_{n}\;.$$

It is possible to fairly compare diverse multiresolution TFRs under the aforementioned standardizations. Here it is assumed a properly conditioned digital signal $x\left(m\right)$, with fast Fourier transform $\hat{x}\left(\omega_{k}\right)=FFT\left\{x\left(m\right)\right\}$, where

(60) $$\omega_{k}=2\pi \frac{k}{M_{L}},\;k=0,\;1,\;2,\ldots M_{L}-1$$

has a substantial duration (>500 points) to make the use of the FFT worthwhile (Appendix B) for the CWT and STX. The total number of points $M_{L}$ should be a power of two,

(61) $$M_{L}=2^{L},\;L\in \mathbb{N},\;L\geq 9,$$

so that the spectral resolution of the FFT for the whole record will be $1/M_{L}$. Note that almost all spectral representations only match predicted theoretical values when the evaluation frequency matches the FFT frequency.

### 2.5. Spectral Entropy

Spectral entropy is a nonlinear transformation that can concentrate energy to improve signal feature extraction e.g., [2]. Like spectral power, it is additive and can be rescaled and normalized in different ways to enhance features associated with sources or physical regimes of interest. Nonlinear transformations are useful for exploratory analyses as well as ML applications. Mallat [15] goes into more detail in the context of convolutional neural networks, which are generally computed using a cascade of linear and nonlinear transformations that can reduce data variability in the direction of local symmetries. An essential part of the signal exploration stage is to identify these local symmetries in cyberspectral space and select methods to enhance features that can provide additional information.

Spectral power, which is in itself a nonlinear quadratic transformation ${\left|S_{tfr}\left(\tau ,\;\omega \right)\right|}^{2}$, is also often reported as a logarithmic amplitude. When the logarithm is of base 10 and the power level is referred to a reference, the resulting nonlinear spectral amplitude is expressed in decibels. In audio and some infrasound applications, the frequencies are also expressed in standardized log frequency bands, e.g. [1]

(62) $$dB_{\tau w}\approx 20\;log_{10}\left|S_{tfr}\left(\tau ,\omega \right)\right|\;.$$

The Shannon spectral information $I_{\tau w}$ of a time–frequency instance is proportional to the frequency marginals

(63) $$I_{\tau w}=-log_{2}\left[P_{tfr}\left(\tau ,\omega \right)\right]$$

and is similar to the dB scale in that represents spectral energy, but it also quantifies information. The instantaneous Shannon spectral entropy can be expressed as

(64) $$H_{\tau w}=-P_{tfr}\left(\tau ,\omega \right)\;log_{2}\left[P_{tfr}\left(\tau ,\omega \right)\right]\;,$$

and has a higher level of nonlinear complexity as well as additive properties that will be discussed in the next section.

The third order Rényi entropy is often invoked by mathematicians to resolve some idiosyncrasies of the Weiner distribution e.g., [9]. However, the final Rényi nonlinear transformation is logarithmic and would only differ from the Shannon information metrics by a scaling constant. The next section develops methods for stabilizing the scaling of power, information, and entropy TFRs to facilitate the extraction of relevant features to be used for signature discovery and identification.

### 2.6. Cyberspectral Information and Entropy

Fast multiresolution TFR methods operate on the FFT of a record. The total entropy of the signal, or the degree of complexity and amount of information in the record, should be stable (within numerical errors) across the diverse methods implemented.

Let $x\left(m\right)$ be a properly conditioned digital input signal with Fourier transform

(65) $$\hat{x}\left(\omega_{k}\right)=\sum_{m=0}^{M-1}x\left(m\right)e^{-j\omega_{k}m}=F\left\{x\left(m\right)\right\}\;.$$

Both the STFT and the Welch periodogram compute the Fourier transform of a smaller window as it slides over specified overlap points; the STFT saves each window, whereas the Welch periodogram sums and averages. The Welch periodogram can be evaluated from the STFT by averaging over all time columns.

Following Williams et al. [9] (Appendix C), one can construct a probability distribution function in the time domain

(66) $$p\left(m\right)=\frac{{\left|x\left(m\right)\right|}^{2}}{\sum_{m=0}^{M-1}{\left|x\left(m\right)\right|}^{2}}$$

(67) $$\sum_{m=0}^{M-1}p\left(m\right)=1$$

and in the frequency domain

(68) $$P\left(k\right)=\frac{{\left|\hat{x}\left(k\right)\right|}^{2}}{\sum_{k=0}^{M-1}{\left|\hat{x}\left(k\right)\right|}^{2}}$$

(69) $$\sum_{k=0}^{M-1}P\left(k\right)=1$$

from the marginals.

In the time domain, the Shannon information is

(70) $$I_{\tau }\left(m\right)=-log_{2}\left[p\left(m\right)\right]$$

and the Shannon entropy is

(71) $$h_{\tau }\left(m\right)=p\left(m\right)\;I_{\tau }\left(m\right)\;.$$

Whereas in the frequency domain, the Shannon information is

(72) $$I_{\omega }\left(k\right)=-log_{2}\left[P\left(k\right)\right]$$

and the Shannon entropy is

(73) $$h_{\omega }\left(k\right)=P\left(k\right)\;I_{\omega }\left(k\right)\;.$$

As previously discussed, the total entropies in the time and frequency domains

(74) $$H_{\tau }\left[p\right]=H_{\tau \;total}=\sum_{m=0}^{M-1}h_{\tau }\left(m\right)\;$$

(75) $$H_{\omega }\left[P\right]=H_{\omega \;total}=\sum_{k=0}^{M-1}h_{\omega }\left(k\right)$$

do not always perfectly match numerically, but they should be close to each other. The total entropy of the system should be stable across all methods.

Each of the instantaneous information and entropy measures are small values, and in order to display them within reasonable bounds it is useful to rescale them relative to a reference value or distribution. A useful information metric for a spectral distribution $P\left(k\right)$ relative to a reference distribution $P_{r}\left(k\right)$, the information signal to noise ratio (ISNR) may be simply expressed in bits as

(76) $$isnr_{\omega }\left[P\left(k\right)||P_{r}\left(k\right)\right]\;\left[\mathrm{bits}\right]=log_{2}\left[\frac{P\left(k\right)}{P_{r}\left(k\right)\;}\right]$$

which can be readily compared to the traditional metric for signal to noise in decibels (dB)

(77) $$snr_{\omega }\left(k\right)\;\left[\mathrm{dB}\right]=10\;log_{10}\left[\frac{P\left(k\right)}{P_{ref}}\right]$$

where $P_{ref}$ is a reference power level.

Entropy algebras are the purview of mathematicians and statisticians [24,25,26,27,28,29,30,31,32], and the nomenclature for entropy divergence metrics can vary substantially. Gray [25] discusses relative entropy metrics in some detail. The general form of the Kullback–Liebler (KL) [26] divergence of distribution *P* relative to $P_{r}$ is

(78) $$D\left(P||P_{r}\;\right)=\sum P\left(k\right)log_{2}\left[\frac{P\left(k\right)}{P_{r}\left(k\right)\;}\right],$$

(79) $$D\left(P||P_{r}\;\right)=\sum P\left(k\right)\;isnr_{\omega }\left[P\left(k\right)||P_{r}\left(k\right)\right]$$

and can also be referred to as the relative entropy, the KL distance, Kullback information, the information gain, and the directed divergence [25,26,27,28,29,30,31,32,33,34,35]. The relative entropy is always greater or equal to zero, only vanishing when the distributions are identical. As mentioned in a study by Ebrahimi et al. [28], the information in a signal refers to the changes induced relative to the probability distribution used for inference. The distribution $P_{r}$ could represent the state of no-signal referred to as noise, a competing signal, or a reference signal. The uniform distribution $P_{r}=U$ is commonly used as the *global reference distribution* [28] for the quantification of change and unpredictability. In the case of the uniform distribution

(80) $$U_{M}\left\{k\in \left[0\right.,\left.M\right)\right\}=1/M$$

(81) $$H_{U_{M}}=H\left[U_{M}\right]=\sum_{k=0}^{M-1}log_{2}\left[M\right]/M=log_{2}\left[M\right]\;.$$

It is worth briefly pausing to consider some of the properties of the total entropy $H_{U_{M}}$ of the uniform distribution. Since

(82) $$H_{U_{M}}=log_{2}\left[M\right]=\sum_{k=0}^{M-1}h_{U_{M}}$$

and the number of points in an input record will always be greater than one, the total entropy of the uniform distribution is always greater than zero and grows with the duration of the record. In contrast, the sample (instantaneous) entropy

(83) $$h_{U_{M}}=log_{2}\left[M\right]/M$$

becomes progressively smaller as the record duration increases,

(84) $$\underset{M\to \infty }{\lim}h_{U_{M}}\to 0\;.$$

Referencing the global uniform distribution, the ISNR simplifies to

(85a) $$isnr_{\omega }\left[P\left(k\right)||U_{M}\right]=log_{2}\left[MP\left(k\right)\right]$$

(85b) $$isnr_{\omega }\left[P\left(k\right)||U_{M}\right]=log_{2}M-I_{\omega }\left(k\right)$$

which can be reduced to

(86) $$isnr_{\omega U}\left(k\right)=H_{U_{M}}-I_{\omega }\left(k\right)$$

resulting in an interesting combination of entropy and information metrics in spectral cyberspace. Alternatively, the ISNR can be estimated from the mean value of the power [2]. Since

(87) $$\sum_{m=0}^{M-1}p\left(m\right)=\sum_{k=0}^{M-1}P\left(k\right)=1,$$

the average value of the marginals can be represented by $\langle p\left(m\right)\rangle =\langle P\left(k\right)\rangle =1/M$.

(88) $$isnr_{\tau 𝓊}\left(m\right)=log_{2}\left[\frac{p\left(m\right)}{\langle p\left(m\right)\rangle }\right]=H_{{𝓊}_{M}}-I_{\tau }\left(m\right)$$

(89) $$isnr_{\omega U}\left(k\right)=log_{2}\left[\frac{P\left(k\right)}{\langle P\left(k\right)\rangle }\right]=H_{U_{M}}-I_{\omega }\left(k\right)\;.$$

Therefore, equivalent relative information metrics are obtained by using the uniform distribution as a reference or the algebraic mean of the power. This returns a very stable and well-scaled instantaneous relative information metric, with units of bits, relative to the degrees of freedom *M* of the distribution. Scaling the entropy is more challenging as the entropy is a nonlinear product that can grow rapidly.

The solution for total relative entropy referenced to a uniform distribution is well known

(90a) $$D\left(P||U_{M}\;\right)=\sum P\left(k\right)\;isnr_{\omega }\left(P\left(k\right)||P_{r}\left(k\right)\right)$$

(90b) $$D\left(P||U_{M}\;\right)=\sum P\left(k\right)\;log_{2}M-\sum P\left(k\right)\;I_{\omega }\left(k\right)$$

where, since $\sum P\left(k\right)=1$

(90c) $$D_{\omega U}=H_{U_{M}}-H_{\omega }\left[P\right]\;.$$

The relative entropy, in bits, is considered a distortion measure that quantifies the distance between the distribution under study and a uniform distribution ([25], Ch. 5). An equivalent relationship can be readily derived for the time marginals

(91) $$D_{\tau 𝓊}=D\left(p||{𝓊}_{M}\;\right)=H_{{𝓊}_{M}}-H_{\tau }\left[p\right]\;.$$

Since the total relative entropy is always greater or equal to zero, the instantaneous relative entropy $d_{\omega U}\left(k\right)$

(92) $$D_{\omega U}=\sum_{k=0}^{M-1}d_{\omega U}\left(k\right)\;$$

can be a small value, and zero only when the distribution is nearly uniform. As with the entropy, it has the convenient property of being additive. It can be compared to the average value of the relative entropy estimated by

(93) $$\langle D_{\omega U}\rangle =\frac{log_{2}M}{M}-\frac{H_{\omega }\left[P\right]}{M}=h_{U_{M}}-\frac{H_{\omega }\left[P\right]}{M}$$

which also yields very small numbers and will change with every signal.

Rearranging Equation (93) yields

(94) $$\frac{h_{\omega }\left(k\right)}{h_{U_{M}}}=1-\frac{d_{\omega U}\left(k\right)}{h_{U_{M}}}\;.$$

A practical scaled entropy metric compresses prominent spectral features into a tractable range. A stable entropy signal to noise ratio (ESNR) can be defined as the ratio of the instantaneous entropy to that of a continuous uniform distribution

(95) $$esnr\left(k\right)=h\left(k\right)/h_{U}\;.$$

It has the advantage of returning unity when the signal distribution resembles a uniform distribution. The more the signal variance deviates from a predictable uniform distribution, the more pronounced the ESNR becomes. In contrast to the ISNR, the ESNR is not in bits but is a ratio of bits, as originally described in ([22], p.13). However, the total and relative entropies, in bits, can be readily recovered from the summed ESNR by multiplying by the scalar constant $h_{U}$

(96) $$H\left[P\right]=h_{U}\sum_{k=0}^{M-1}esnr\left(k\right)=H_{U}-D\left[P||U\right].$$

The information SNR is in bits and can be expressed as

(97) $$isnr\left(k\right)=H_{U}-I\left(k\right)\;.$$

These new relations are remarkably stable as they scale with the population size. The expected value of information, entropy, is traditionally provided as total quantity over an integration time and/or bandwidth. Traditional entropy studies provide a single number for the duration or bandwidth of a signature of interest. In the next section, spectral information and entropy SNRs are further developed to quantify these metrics at the same resolution as the original time–frequency representation, inviting high-resolution exploration of signature features otherwise occluded by the averaging.

## 3. Algorithm Evaluation and Results

The scope of this paper swerved sharply on 15 January 2022, when a volcanic eruption in Tonga produced the largest explosion of the century, exciting Earth oscillations observed by diverse digital sensor systems in unsurpassed detail and scope. Two of many e.g., [47,48] feature-rich broadband pressure signatures are selected to illustrate the methods in this paper and lay a foundation for future exploratory studies.

### 3.1. Broadband Transient Signal: Tonga Lamb Waves

Propagating pressure waves emitted during the ~200 megaton [47] Hunga Tonga-Hunga Ha’apai volcano eruption circumnavigated Earth multiple times. The most prominent waveform signature, referred to as the Tonga Lamb wave, is an exceptional example of a broadband transient signal. In this paper, signals are considered encoded messages containing information about a source. The propagation of that signal to a digital cyber-physical measurement system is treated as the communication of that information through a transmission channel. In this context, the Lamb wave transmitted eruption source information globally through multiple atmospheric wave regimes including (but not limited to) hydrodynamic, internal, acoustic-gravity, and infrasonic perturbations from ground to space [47,48].

The first time series selected for this stage of the case study is from International Monitoring System (IMS) infrasound Station I22 in Port Laguerre, New Caledonia, located ~2000 km from the blast epicenter [47]. The preprocessed data have a sample rate of 1 Hz and have been instrument corrected down to the microhertz (μHz) range. The New Caledonia station provides a valuable representative signal as it is the closest IMS infrasound array to the source epicenter, and is likely to be a baseline for future studies.

**Figure 1:**
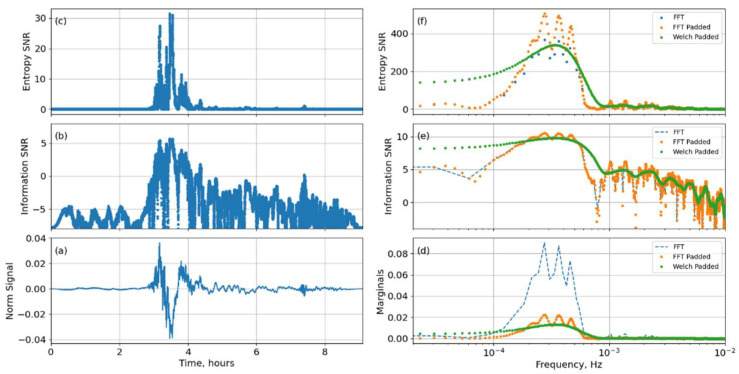
Time (**a**–**c**) and frequency (**d**–**f**) representations of the signal observed by an IMS infrasound station in New Caledonia. The dotted blue line represents the FFT of the input signal, the orange dots represent the FFT of the zero-padded signal, and the green line the Welch periodogram of the zero-padded STFT subwindow with a high overlap.

The I22 signal consists of a microbarometric pressure record with $M_{L}=2^{15}\;$sample points, or ~9.102 h (9 h 6 m 8 s), and is shown in Figure 1a. The nominal period of the primary Tonga Lamb wave is ~500 μHz (2000 s~33 min). Ideally, the averaging frequency is at least a couple of octaves below the target frequency, and an averaging frequency of 100 μHz is selected for the multiresolution computations. There is a secondary, less energetic Lamb emitted wave ~4 h after the primary signal [47]; although the next analyses are tuned to the primary Lamb wave, both signatures are of interest.

From Section 2.4.5, a third octave specification for the averaging Gabor atom returns $M_{J}=2^{17}>M_{L}$, requiring zero padding of the input signal. The STFT sliding window presents an additional requirement, as it must always be smaller than the input signal duration. Two cycles of the target period can be captured with a window duration of 4096 s (~1 h 8 m period) and can be represented with an FFT duration of $M_{FFT}=2^{12}=M_{L}/8\;$, with a marginal spectral resolution of 250 μHz without zero padding. Such a coarse spectrogram would need a high spectral overlap to be somewhat comparable to multiresolution TFRs.

The I22 input signal shown in Figure 1 captures the climactic stage of the Tonga eruption and the emission of Lamb waves. The left three panels of Figure 1 shows time domain representations; in panel (a), the input signal has been normalized by the rms power, so its square returns the time marginals $p\left(m\right)$, (b) shows the ISNR, and (c) shows the ESNR.

The equations for the ordered dyadic framework described in Section 2.4.5 are used to determine the key spectral parameters. The right panel of Figure 1 shows the frequency domain representations with (d) marginals from the FFT coefficients, (e) ISNR, and (f) ESNR for an input signal duration of $M_{L}=2^{15}\;$sample points (blue). A zero-padded signal duration of $M_{J}=2^{17}>M_{L}$ corresponding to an averaging frequency of 0.1 mHz, is shown as orange dots. The Welch periodogram duration (which would match the STFT sliding window duration) must be substantially shorter than the non-padded record duration. A STFT/Welch duration of $M_{stft}=2^{12}=M_{L}/8\;$(zero padded to $M_{J}=2^{17}$) is shown as green dots. The zero-padding can be recognized as an efficient way of interpolating in the frequency domain, and is built into most FFT-based algorithms (*nperseg* =$\;\;2^{17}$, *nfft* = $2^{12}$ in [43]). However, the degraded spectral resolution from the smaller Welch/STFT window is expected and evident, as is the spectral smoothening resulting from the mean Welch averaging.

#### 3.1.1. Evaluation of the Short Time Gabor Transform (GTX)

The GTX is a STFT with a Gaussian window, and the same parameters as for the Welch periodogram in Figure 1d–f are used. The GTX specifications are the input record duration $M_{L}$, the sliding Gaussian-tapered FFT window duration $M_{gtx}$, and the zero-padded window duration $M_{J}$,

(98) $$M_{gtx}=2^{12},M_{L}=2^{15},\;M_{J}=2^{17}\;.$$

As mentioned in Section 3.1, these specifications would produce a very coarse spectrogram without overlapping windows. A 95% window overlap specification will yield substantial oversampling and spectral leakage in the TFR, but it will allow comparisons with the multiresolution methods. As discussed, the positive angular frequencies of the GTX are

(99) $$\omega_{k}=2\pi \frac{m}{M_{J}},\;m=0,\;1,\;2,\ldots M_{J}/2\;.$$

It is well known that a tone of unit amplitude will have a variance of ½, where this variance is distributed over the positive and negative frequency axes. Since only the positive frequencies are evaluated, a factor of two is needed to recover the variance:

(100) $$P_{gtx}\left(\tau ,\omega \right)=2{\left|S_{gtx}\left(\tau ,\omega \right)\right|}^{2}\;.$$

The SciPy [43] spectrogram function is employed using a Gaussian window with a standard deviation of $M_{gtx}/4$. The resulting spectrograms were nearly identical to those computed with the default Hann window.

#### 3.1.2. Evaluation of the Continuous Wavelet Transform (CWT)

The CWT does not have a hop length as it operates over the complete record. As outlined in Section 3.1, the CWT specifications for third octave bands (N=3) and an averaging frequency of 100 μHz are

(101) $$M_{J}=2^{17}\;.$$

The positive FFT frequencies are

(102) $${\tilde{f}}_{k}=\frac{k}{M_{J}},\;k=0,\;1,\;2,\ldots M_{J}/2\;.$$

The Gabor atom frequencies are evaluated at standard fractional octave bands between the averaging and Nyquist frequencies. Nyquist sets the smallest scale, with band number $n_{min}$. The averaging scale is the lowest frequency of interest, and should be at least an octave below the center frequency. As mentioned in the previous paragraph, the averaging frequency is set by$\;M_{J}$, which sets the upper (largest) scale band number $n_{max}$.

For the selected input signal, $\;{\tilde{f}}_{s\;}={\tilde{f}}_{p0}=1$, and let G=2 and $M_{J}=2^{J}$

(103) $${\tilde{f}}_{n}=2^{-\frac{n}{N}}$$

(104) $$n_{nyq}<n\leq n_{max}$$

(105) $$n_{nyq}=N\;\mathrm{(Nyquist)}$$

(106) $$n_{max}=\mathrm{floor}\left[N\left\{\;J-log_{2}M_{N}\right\}\right]=N\;J-ceil\left(Nlog_{2}M_{N}\right)$$

where

(107) $$N\;log_{2}M_{N}=N\;log_{2}\left[\frac{3\pi }{4}N\right]\;.$$

Although these expressions are cumbersome in writing, they are straightforward to implement in code. For N=3, $M_{J}=2^{17}$

(108) $${\tilde{f}}_{n}=2^{-\frac{n}{3}}$$

(109) $$n_{min}=n_{nyq}+1,\;\;\;n_{min}\leq n\leq n_{max}\;.$$

Special care should be taken to match the FFT and center frequencies if accurate signal power reconstruction is a requirement. For a real signal

(110) $$P_{cwt}\left(\tau ,\omega \right)=2{\left|S_{cwt}\left(\tau ,\omega \right)\right|}^{2}\;.$$

#### 3.1.3. Evaluation of the Stockwell Transform (STX)

As with the CWT, the STX does not have a hop length as it operates over the complete record. The STX specifications are as with the CWT

(111) $$M_{L}=2^{15},\;M_{J}=2^{17}\;.$$

with positive FFT frequencies

(112) $${\tilde{f}}_{k}=\frac{k}{M_{L}},k=0,\;1,\;2,\ldots M_{L}/2\;.$$

As with the CWT, Gabor atom frequencies can be evaluated at standard fractional octave bands. The Nyquist frequency sets the smallest possible scale, with band number $n_{min}$. The averaging scale is set by$\;M_{J}$, which defines the upper (largest) scale band number $n_{max}$. It is also possible to construct time–frequency representations using alternate frequency spacing schemas and specifications such as melody (mel) frequencies or linearly spaced frequency bands for direct comparison with STFT results. This is also viable with the CWT, leading to the possibility of severely underdetermined or overdetermined energy estimates.

The numerical recipe for evaluating the Stockwell transform is well known and documented. For each Stockwell frequency $\omega_{n}$, construct the matrix

(113) $$z\left(t,\;\omega_{n}\right)=x\left(t\right)\;e^{-j\omega_{n}t}$$

and take its FFT,

(114) $$\hat{z}\left(\omega_{k},\omega_{n}\right)=\mathrm{FFT}\left[x\left(t\right)\;e^{-j\omega_{n}t}\right]=\hat{x}\left(\omega_{k}+\omega_{n}\right)\;.$$

Then, multiply each frequency component by the Fourier transform of the Gabor atom

(115) $${\hat{g}}_{n}\left(\omega_{k},\omega_{n}\right)=exp\left\{-\frac{1}{2}{\left[\omega_{k}\sigma_{n}\right]}^{2}\right\}$$

where

(116) $$\sigma_{n}=\frac{9\pi }{\omega_{n}},\;N=3$$

(117) $$\omega_{n}=2\pi {\tilde{f}}_{n}\;.$$

The Stockwell matrix is the inverse FFT (IFFT) of the product over the $\omega_{k}$ frequencies

(118) $$S_{stx}\left(t,\omega_{n}\right)=\mathrm{IFFT}\left\{{\hat{z}}_{n}{\hat{g}}_{n}\left(\omega_{k},\omega_{n}\right)\right\}\;.$$

The spectral power density is estimated from

(119) $$P_{stx}\left(\tau ,\omega \right)=2{\left|S_{stx}\left(\tau ,\omega \right)\right|}^{2}$$

as with $P_{cwt}.$

### 3.2. Cyberspectral Information and Entropy

Three different methods for constructing spectral representations for relative information and entropy are presented. They provide alternatives to traditional spectrograms and are specifically designed for enhancing cyberspectral information in transient signatures for feature discovery, extraction and further signal processing for pattern recognition.

#### 3.2.1. Grid Distribution

The most common and intuitive methods for scaling information and entropy use the selected time and bandpass of the TFR to construct the energy distribution from the spectral power time–frequency grid. This approach was discussed in [2] to introduce an entropy signal to the noise ratio (SNR). The concept builds on the additive property of energy and entropy. The total spectral energy in a TFR can be computed from the sum of the spectral matrix time–frequency grid at each time *m* and frequency *n*

(120) $$P_{G}=\sum_{m=0}^{M_{L}-1}\sum_{n=n_{min}}^{n_{max}}P_{m,n}$$

with scaled power

(121) $${\hat{P}}_{m,n}=P_{m,n}/P_{G}$$

to satisfy

(122) $$\sum_{m}\sum_{n}{\hat{P}}_{m,n}=1\;.$$

An average power per time–frequency grid point can be defined as

(123) $${\hat{P}}_{G}=\frac{1}{M_{L}N_{B}}\sum_{m}\sum_{n}{\hat{P}}_{m,n}=\frac{1}{M_{L}N_{B}}\;,$$

where $N_{B}$ is the number of frequency bands

(124) $$N_{B}=n_{max}-n_{min}+1\;.$$

Since the information per time–frequency grid point is

(125) $$I_{m,n}=-log_{2}{\hat{P}}_{m,n}$$

a spectral SNR can be defined relative to the global grid average

(126) $$snr_{G,m,n}={\hat{P}}_{m,n}/{\hat{P}}_{G}=M_{L}N_{B}{\hat{P}}_{m,n}$$

and a grid ISNR can be represented by

(127a) $$isnr_{G,m,n}=log_{2}\left(snr_{G,m,n}\right)=log_{2}\left(M_{L}N_{B}\right)+log_{2}\left({\hat{P}}_{m,n}\right)$$

(127b) $$isnr_{G,m,n}=log_{2}\left(M_{L}\right)+log_{2}\left(N_{B}\right)-I_{m,n}\;.$$

This simple ISNR is directly proportional to the information per grid point and can be expressed in bits, and, as demonstrated in Section 2.6, it is equivalent to using a normal distribution as the global reference. The ISNR is a useful metric for displaying the global extrema in logarithmic amplitude scales and is comparable to the SNR in decibels relative to any other distribution, be it a reference signal or a noise model. The information relative to the reference distribution would be expressed as

(128) $$snr_{m,n}\left(P||Q\right)={\hat{P}}_{m,n}/{\hat{P}}_{r;\;m,n}$$

(129) $$isnr_{G,m,n}\left(P||Q\right)=log_{2}\left({\hat{P}}_{m,n}/{\hat{P}}_{r;\;m,n}\right)\;,$$

where ${\hat{P}}_{r;\;m,n}$ is the distribution representative of a noise model or a competing signal, where it is implicit that one person’s noise is another person’s signal. The two-dimensional KL divergence [26] follows the one-dimensional case outlined in Section 3.1.1.

The entropy at each grid point is

(130) $$h_{m,n}=-{\hat{P}}_{m,n}log_{2}{\hat{P}}_{m,n}={\hat{P}}_{m,n}I_{m,n}$$

with total grid entropy

(131) $$H_{G}=\sum_{m=0}^{M_{L}-1}\sum_{n=n_{min}}^{n_{max}}h_{m,n}\;.$$

This is a measure of the expected value of information in the TFR. Most traditional spectral entropy studies would stop here, returning a single Shannon entropy value in the cyberspace canvas. An additional transformation returns a stable instantaneous ESNR. Relative to a uniform distribution where the probability is constant

(132) $${\hat{P}}_{r;\;m,n}=U_{M_{L}N_{B}}=\frac{1}{M_{L}N_{B}}\;,$$

the instantaneous reference entropy is

(133) $$h_{U}\left(m,n\right)=\frac{1}{M_{L}N_{B}}log_{2}\left(M_{L}N_{B}\right)$$

which is used to scale the ESNR relative to the uniform distribution

(134) $$esnr_{G,m,n}=\frac{h_{m,n}}{h_{u}}\;.$$

The partial Shannon entropy in bits in a subset of the spectral cyberspace is the sum over the selected time and bandwidth of interest, multiplied by the reference entropy.

#### 3.2.2. Time Distribution

Another familiar method of scaling time–frequency energy is per frequency band [23]. This is reminiscent of the approach of [9], but at the signal level. In this approach, the distribution is computed from the energy in the cyberspectral matrix at each frequency *n,*

(135) $$PF_{n}=\sum_{m=0}^{M_{L}-1}P_{m,n}$$

with scaled power

(136) $${\hat{PF}}_{m,n}=P_{m,n}/PF_{n}\;.$$

It satisfies

(137) $$\sum_{m}{\hat{PF}}_{m,n}=1$$

(138) $$\sum_{m}\sum_{n}{\hat{PF}}_{m,n}=N_{B}\;.$$

The information per frequency band is

(139) $$IF_{m,n}=-log_{2}{\hat{PF}}_{m,n}\;,$$

and the Shannon information per band is

(140) $$hF_{m,n}=-{\hat{PF}}_{m,n}log_{2}{\hat{PF}}_{m,n}={\hat{PF}}_{m,n}IF_{m,n}$$

with reference value

(141) $$hF_{U}=\frac{1}{M_{L}}log_{2}\left(M_{L}\right)\;.$$

The information SNR per frequency band at each grid point can be represented by

(142) $$isnr_{F,m,n}=log_{2}\left(M_{L}\right)-IF_{m,n}\;,$$

and the entropy SNR per frequency band at each grid point can be represented by

(143) $$esnr_{F,m,n}=\frac{hF_{m,n}}{hF_{U}}\;.$$

#### 3.2.3. Frequency Distribution

Another familiar method of scaling time–frequency energy is across all frequency bands e.g., [23], where the distribution is computed from the spectral energy in the time–frequency grid at each time step *m.*

(144) $$PT_{n}=\sum_{n=n_{min}}^{n_{max}}P_{m,n}$$

with scaled power

(145) $${\hat{PT}}_{m,n}=P_{m,n}/PT_{n}$$

and satisfies

(146) $$\sum_{n}{\hat{PT}}_{m,n}=1$$

(147) $$\sum_{m}\sum_{n}{\hat{PT}}_{m,n}=M_{L}\;.$$

The information per time step is

(148) $$IT_{m,n}=-log_{2}{\hat{PT}}_{m,n}$$

and the Shannon information per time step is

(149) $$hT_{m,n}=-{\hat{PT}}_{m,n}log_{2}{\hat{PT}}_{m,n}={\hat{PT}}_{m,n}IT_{m,n}$$

with reference value

(150) $$hT_{U}=\frac{1}{N_{B}}log_{2}\left(N_{B}\right)\;.$$

The ISNR per time can be represented by

(151) $$isnr_{T,m,n}=log_{2}\left(N_{B}\right)-IT_{m,n}$$

and the ESNR per time can be represented by

(152) $$esnr_{T,m,n}=\frac{hT_{m,n}}{hT_{U}}\;.$$

#### 3.2.4. Short-Time Fourier Transform (STFT) Information and Entropy

Special care must be taken near the edges of a tapered window when computing the information SNR per frequency to prevent division by zero. The GTX ISNR and ESNR are as for the previous cases. However, as suggested by Figure 1, it is possible to overcount the number of frequencies. In contrast to the CWT and STX, which are evaluated within clearly specified passbands, the GTX computes all the frequencies starting at zero. It may be useful to re-scale the GTX entropy by the passband under consideration. Possible ESNR scaling alternatives are briefly discussed in Section 4. Due to the coarseness of the GTX window, a 95% overlap is used to improve the presentation of the GTX TFRs at the expense of substantial computational redundancy in both the time and frequency domains.

### 3.3. Results

**Figure 2:**
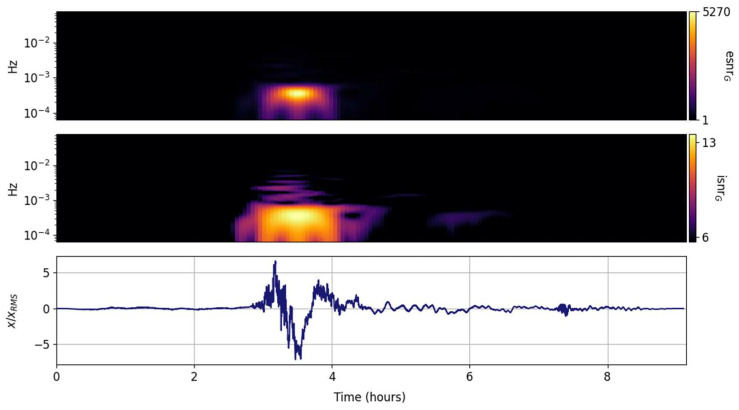
Input signal (**lower panel**) scaled by its rms and its GTX grid information snr ($isnr_{G,m,n}$, **middle panel**) and entropy snr ($esnr_{G,m,n}$, **upper panel**).

**Figure 3:**
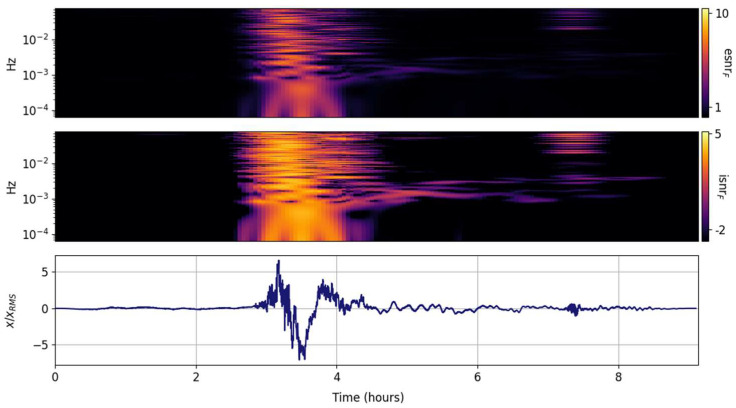
Scaled input signal (**lower panel**) and its GTX information snr ($isnr_{F,m,n}$, **middle panel**) and entropy snr ($esnr_{F,m,n}$, **upper panel**) per frequency band.

**Figure 4:**
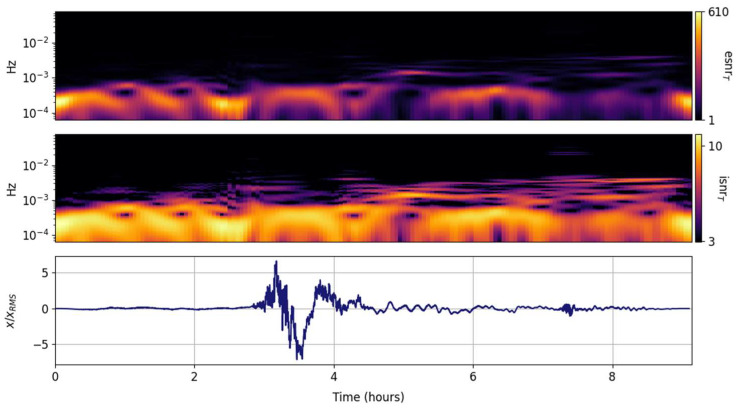
Scaled input signal (**lower panel**) and its GTX information snr ($isnr_{T,m,n}$, **middle panel**) and entropy snr ($esnr_{T,m,n}$, **upper panel**) per time step.

**Figure 5:**
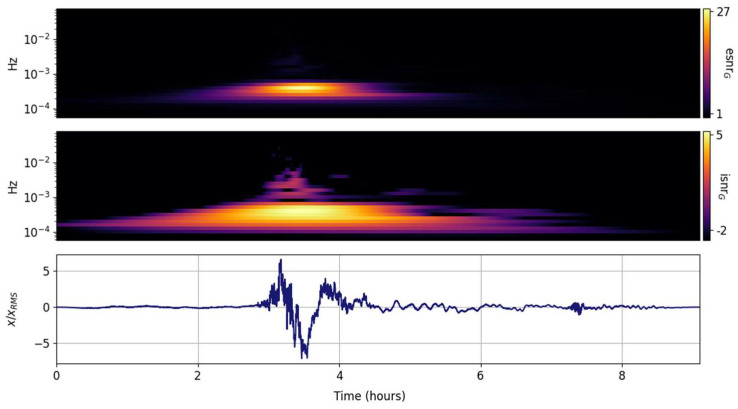
Input signal (**lower panel**) and its CWT grid information snr ($isnr_{G,m,n}$, **middle panel**) and entropy snr ($esnr_{G,m,n}$, **upper panel**).

**Figure 6:**
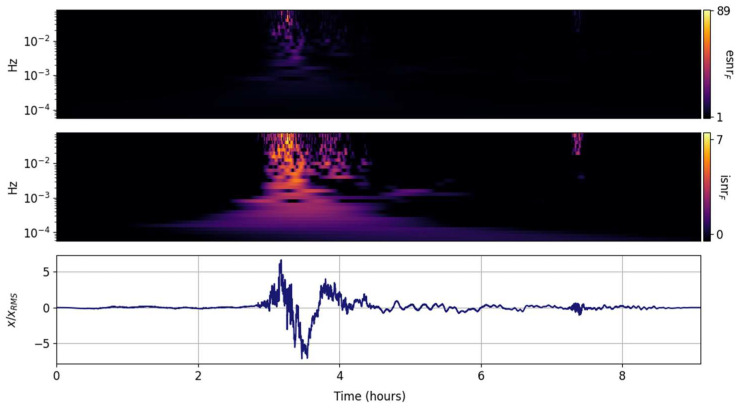
Scaled input signal (**lower panel**) and its CWT information snr ($isnr_{F,m,n}$, **middle panel**) and entropy snr ($esnr_{F,m,n}$, **upper panel**) per frequency band.

**Figure 7:**
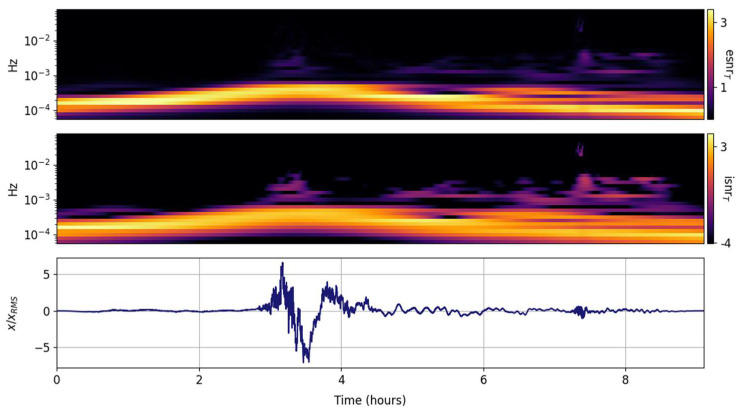
Scaled input signal (**lower panel**) and its CWT information snr ($isnr_{T,m,n}$, **middle panel**) and entropy snr ($esnr_{T,m,n}$, **upper panel**) per time step.

**Figure 8:**
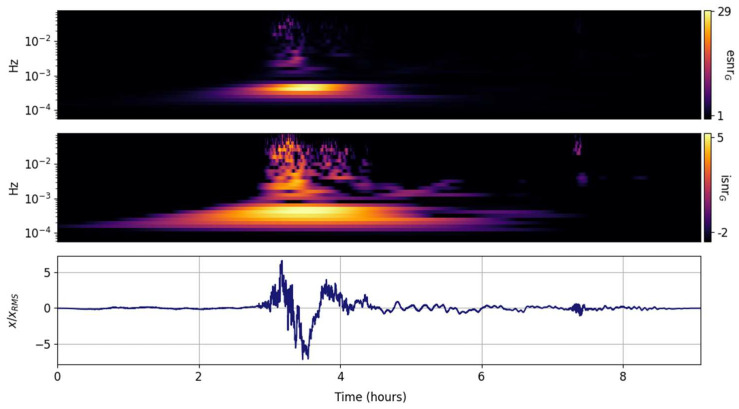
Input signal (**lower panel**) and its STX grid information snr ($isnr_{G,m,n}$, **middle panel**) and entropy snr ($esnr_{G,m,n}$, **upper panel**).

**Figure 9:**
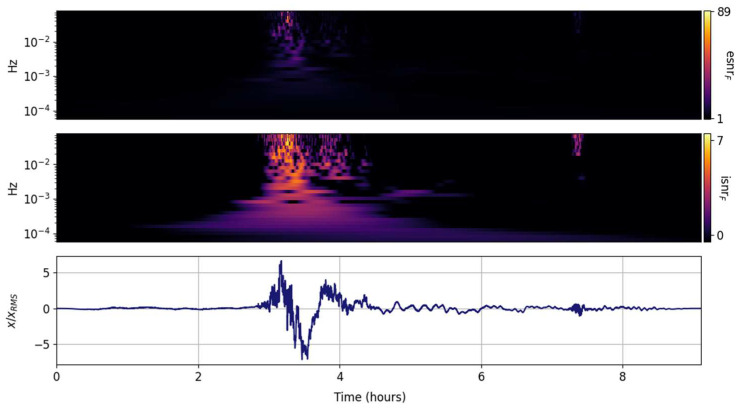
Scaled input signal (**lower panel**) and its STX information snr ($isnr_{F,m,n}$, **middle panel**) and entropy snr ($esnr_{F,m,n}$, **upper panel**) per frequency band.

**Figure 10:**
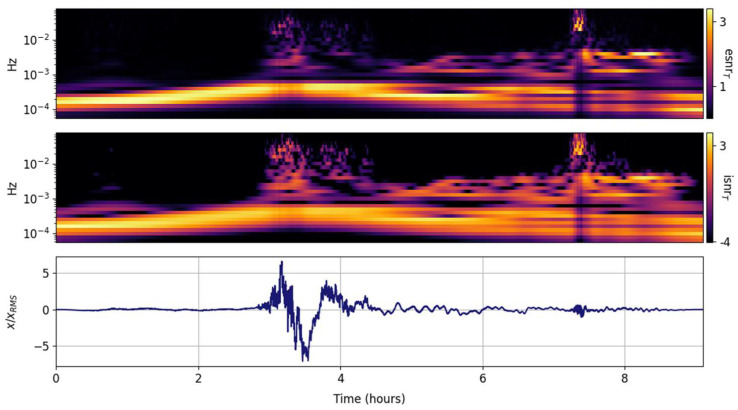
Scaled input signal (**lower panel**) and its STX information snr ($isnr_{T,m,n}$, **middle panel**) and entropy snr ($esnr_{T,m,n}$, **upper panel**) per time step.

The spectral ISNR and ESNR are shown in Figure 2, Figure 3, Figure 4, Figure 5, Figure 6, Figure 7, Figure 8, Figure 9 and Figure 10 for the STFT with a Gaussian window (GTX), the quantized continuous wavelet transform (CWT), and the quantized Stockwell transform (STX). The TFR input parameters are as described in Section 3.1 and are tuned to the primary Lamb wave.

#### 3.3.1. Gabor Transform (GTX)

The temporal issues with the STFT and its taper-windowed variants are well known and documented; although exceptionally well suited for continuous-wave tones or slowly varying sweeps, it often fails to capture drastic spectral changes in many transients. The GTX transform of the Tonga Lamb wave is expected to fail, and is presented here as a baseline and motivation to consider alternate spectral transformations that can be tuned to the signal of interest. The figures presented in this section all have the same structure; the lower panel is the input time series, the middle panel shows the information SNR, and the upper panel shows the entropy SNR. In contrast to the time series in Figure 1, which is scaled by the square root of the total power, the time series in Figure 2, Figure 3, Figure 4, Figure 5, Figure 6, Figure 7, Figure 8, Figure 9 and Figure 10 are scaled by the root mean square to highlight the regions that exceed the mean power. Figure 2 shows the spectral information and entropy grid SNRs. Rapid changes in the signal spectrum are averaged out and result in the suppression of high frequency information that would otherwise be prominent (see [47]). In contrast to the CWT and the STX, which have the same temporal granularity as the input record, the GTX time steps are coarse and determined mostly by the 95% overlap.

The spectral ISNR and ESNR distributions per frequency band are shown in Figure 3. Since every frequency band is equally weighted (in contrast to the global weighting), the temporal localization of the peak energy per band is enhanced. Tragically and predictably, the temporal resolution of the GTX is fixed and coarse, returning temporally smeared spectra. The second, smaller Lamb wave before the 8 h mark begins to emerge.

Spectral ISNR and ESNR per time step are shown in Figure 4. Although the distribution population scales with the number of FFT points, the spectral smoothing will be comparable to the one shown by the green line in Figure 1d–f. One thing that is evident in this and the next $esnr_{T}$ distributions is that they effectively highlight the distribution of energy around the main signal. The ambient noise before the Tonga Lamb wave is substantial and in the same internal wave band (<1 mHz) as the main signal. In this GTX representation, both the primary and secondary Lamb waves are small perturbations amidst the ambient noise of Earth’s atmosphere. This is expected; STFT variants are preferentially tuned to persistent continuous wave oscillations.

#### 3.3.2. Continuous Wavelet Transform (CWT)

The multiresolution CWT TFR is expected to outperform the GTX for rapidly changing transients. The compromises are well known; the increased temporal resolution at high frequencies with decreased temporal resolution at low frequencies, and coarser but well-constrained logarithmically spaced frequency bands for the quantized Gabor atoms [2]. The primary Lamb wave energy and its roll off with increasing frequency are well captured in Figure 5. As expected, the CWT convolution acts as a smoothening filter and the second Lamb wave is barely visible in the grid TFRs on Figure 5. The value of the nonlinear ESNR representation is highlighted in the upper panel of Figure 5; only the dominant spectral component is clearly highlighted and could be readily and unambiguously extracted by the maximum of the grid. Since the temporal resolution of the CWT TFR is the same as the input signal, it is possible to temporally localize the spectral peak maximum within a standardized frequency band. The time and frequency uncertainty of the solution is known, well constrained, and its product is minimal and constant.

The CWT spectral ISNR and ESNR per frequency band are shown in Figure 6. As with the GTX, the temporal localization of the peak energy per band is enhanced and the second, smaller, shorter period Lamb wave emerges. In contrast to the GTX, the temporal and spectral resolution are high. In many ways, the $esnr_{F}$ behaves as a highpass filter.

Spectral ISNR and ESNR per time step are shown in Figure 7 for the CWT. The distribution population scales with the number of CWT frequency bins, which may be sparse for low orders and bandwidth. As with the GTX $esnr_{T}$ distribution, it effectively highlights the distribution of ambient noise energy. Although both the primary and secondary Lamb waves are present as small transients in Earth’s ambient noise, they are more prominent than in the GTX. This representation shows an intriguing trend: spectral energy levels pitch up with the arrival of the primary Lamb wave and then broaden afterwards with the emission of diverse wave types associated with the ensuing powerful eruption.

#### 3.3.3. Stockwell Transform (STX)

The multiresolution TFRs with the STX outperform the GTX and the CWT for the selected signature. Although they share the same resolution and uncertainty specifications as the CWT, the release of the phase translation in the STX provides some notable enhancements. The primary Lamb wave energy and its rolloff with increasing frequency are well captured, with less convolution smoothening than the CWT; the second Lamb wave is now much more visible in the grid ISNR on Figure 8. The dominant spectral component is still clearly highlighted in the ESNR and could be readily extracted and represented by the most informative atom coefficients in the grid [2]. As with the CWT, it is possible to temporally localize the spectral peak maximum within a frequency band with a minimal time–frequency uncertainty product.

The STX spectral ISNR and ESNR per frequency band are shown in Figure 9, and are nearly identical to the CWT. As mentioned above, in many ways the $esnr_{F}$ behaves as a highpass filter, suggesting the CWT and STX methods converge at high frequencies.

In contrast, the STX spectral ISNR and ESNR per time step shown Figure 10 contain more fine structure information than those of the CWT (Figure 7). The STX $esnr_{T}$ enhances the instantaneous redistribution of energy of the ambient noise. Both the primary and secondary Lamb waves show up clearly. The STX $esnr_{T}$ representation reinforces the CWT narrative: spectral energy concentration increases in frequency with the arrival of the primary Lamb wave and then broadens in bandwidth with the radiation of a more complex wavefield.

Although the STX representation in Figure 8 is similar to and inspired by the Stockwell transform shown by Vergoz et al. [47], the context is different. Through a combination of standardized, transportable, and reproducible linear and nonlinear operations, the geophysical pressure signature is transformed into three different instantaneous information and entropy distributions to highlight different aspects of the signal and its ambient noise. The transformation to information unveils otherwise hidden spectral features, thereby increasing the number of choices available for exploratory signal analysis and pattern recognition. What we do with the increased amount of information is up to our imagination.

#### 3.3.4. Spectral STX Entropy for New Caledonia Relative to Tonga

The New Caledonia station it is the closest IMS infrasound array to the source epicenter. However, data from closer stations is available and has been discussed elsewhere e.g., [47,48]. One of many interesting questions about this event is how accurately was source information transmitted from Tonga to New Caledonia. This is only a representative example of the many possible studies that can be pursued with these new metrics.

Fortunately, there was barometric pressure data collected in Tonga by the Australian Bureau of Meteorology [47,48,49]. A weather station in Nukuʻalofa (NUKU), Tonga, ~70 km away from the eruption epicenter, sampled absolute pressure every 60 s (Nyquist of 120 s) and reported an unexpected peak Lamb wave polarity. The observed inverted gauge pressure does not appear to be an instrumental artifact. The NUKU station is described in some detail in [49], which hypothesizes that the observed pressure signal could be explained, to first order, by near-source hydrodynamic effects. However, this is not a geophysics paper; more detailed fluid dynamic investigations will be addressed in future work. The main question addressed here is whether the Lamb wave signature in New Caledonia is sufficiently similar to the Tonga signature that the two may be associated. All station data have been time shifted to account for propagation to the station at the nominal Lamb wave speed of 310 m/s. It is worth noting that the two selected stations use different types of pressure sensors; NUKU is a meteorological barometer, I22 uses an infrasonic sensor that has been instrument-corrected to overlap within the Lamb wave bandpass [47].

**Figure 11:**
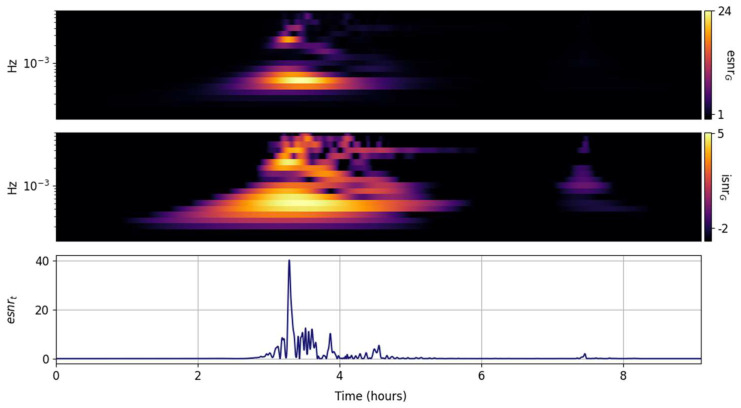
Station NUKU, Tonga. Scaled time-domain entropy snr ($isnr_{t}$, **lower panel**) and its STX grid information snr ($isnr_{G,m,n}$, **middle panel**) and entropy snr ($esnr_{G,m,n}$, **upper panel**) up to 120 s period (8 mHz).

**Figure 12:**
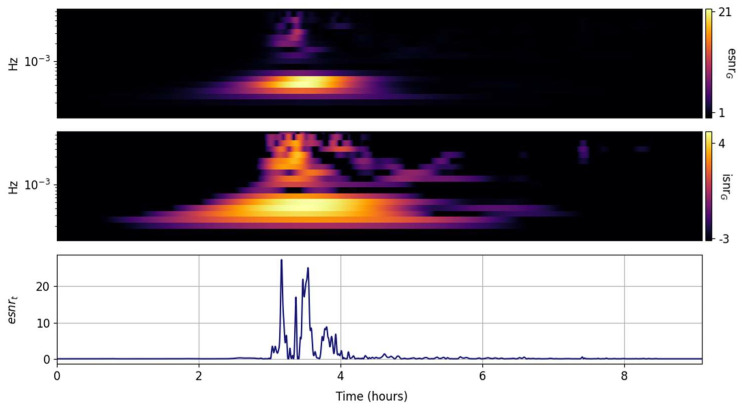
Station I22, New Caledonia. Low-passed filtered scaled time-domain entropy snr ($isnr_{t}$, **lower panel**) and its STX grid information snr ($isnr_{G,m,n}$, **middle panel**) and entropy snr ($esnr_{G,m,n}$, **upper panel**) up to 8 mHz.

The lower panel in Figure 11 shows the instantaneous scaled entropy SNR in the time domain (Figure 1) for the NUKU station in Tonga, as well as the grid ISNR and ESNR up to the 120 s Nyquist period (~8 mHz). Figure 12 shows the same quantities for the I22 station in New Caledonia as in Figure 8, but low-passed filtered below 8 mHz.

The entropy of both signals peaks at the same time-corrected sample. Therefore, the simple travel time correction is sufficient to validate that the signals are causally related. In other words, the time difference between the arrival of the waves in Tonga and New Caledonia support a dominant signal propagating at the nominal theoretical Lamb wave speed of 310 m/s.

A cursory inspection of Figure 11 and Figure 12 shows the two signals have very similar spectral information and entropy distributions. To the author’s knowledge, this is the first time this association has been presented. Thus temporal causality has been verified by the travel time corrections, and spectral association is clear in the time–frequency representations. This reinforces the notion that the two stations observed the same source process. The total Shannon entropy over the grid is ~16.19 bits at Tonga and ~16.25 bits at New Caledonia, a barely distinguishable difference. The two signals have comparable expected values of information despite the substantial distance from each other. The combination of the travel times with the similar expected values and distributions of spectral information provides convincing evidence that the two stations are receiving the same message transmitted by the Lamb wave.

**Figure 13:**
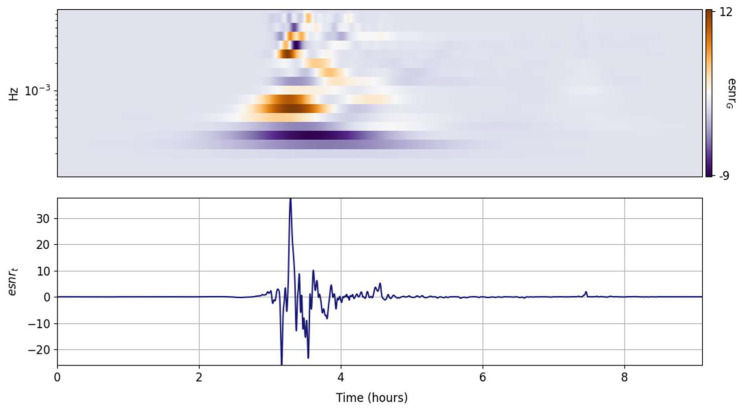
Entropy loss between New Caledonia and Tonga. Time-domain entropy loss ($isnr_{t}$, **lower panel**) and its STX grid entropy snr ($esnr_{G,m,n}$, **upper panel**) computed from the difference of the results shown in Figure 11 and Figure 12.

Most traditional entropy studies would stop at this stage. However, it is also of interest to quantify how information is redistributed along the transmission line. Since the peak in the temporal ESNR is at the same corrected time for both stations, the entropy difference quantifies instantaneous entropy loss in the propagation from Tonga to New Caledonia (~2000 km away). Figure 13 shows the scaled entropy loss, illustrating when, where and how much spectral information is redistributed during transmission. A positive value is loss, whereas a negative value is gain. Energy appears to have been transferred from the higher frequencies to lower frequencies during transmission, which could be associated with the transition from near-source hydrodynamic flow to far-field Lamb wave propagation [49]. Note that the secondary Lamb wave has diminished energy for periods longer than 120 s, and is only clearly visible in the Tonga station in that passband.

The spectral entropy loss shown in Figure 13 is not a typical data product. Propagation loss is traditionally displayed in decibels relative to a geophysical domain-specific reference, which is generally only comparable amongst the same sensor and data types. In contrast, entropy loss is evaluated relative to the energy distribution, and it is conceptually possible to compare dissimilar sensor types in the same bandwidth. The oscillatory nature of instantaneous time-domain entropy loss is intriguing, and may contain valuable information when applied to other stations in this global data set. Oscillatory loss metrics can also be expected from $esnr_{T}$ and $isnr_{F}$ and could be considered in future work.

It is anticipated that more detailed future investigations and data products will yield substantially more insight than this simple case study. Quantifying the temporal and spectral variance between the thousands of stations that recorded this signature throughout Earth will be a topic of study for generations to come, and outside the scope of this paper.

## 4. Discussion

Information is a polysemantic concept; its practical meaning is connected to intent [23]. The intent of this work was to quantify relative information for time-frequency distributions at a granular level. Its goals were to mature the relative spectral information and entropy concepts within a standardized constant-Q Gabor atom framework [2]. The new methods introduce stable, high-resolution tools that can be readily used for signal exploration. This paper is content-rich, and some of the more important contributions are summarized.

Equations (12)–(14) for the compact support of a Gabor logon of fractional order are some of the most succinct and important expressions in this paper, as they contain the spectral energy and time–frequency uncertainty within constant-Q bands. Abiding by these equations reduces the probability of either under- or over-specifying the time–frequency support of the logon, thereby stabilizing it for automation at scale. The author has not found a comparably concise expression in the literature.

With order and standardized frequencies as organizing principles, the quantized Gabor atom permits the construction of complete, transportable, and reproducible spectral cyberspaces with minimal uncertainty. The relations in Section 2.4.5 facilitate the computation of sensor-agnostic spectral cyberspaces to match standardized, constant-Q physical frequencies for signal exploration, feature selection, and data fusion [2,37]. These expressions, and their value for facilitating sensor transportability, are easy to miss.

The relative Shannon spectral information and entropy were computed for selected time series of geophysical interest, using three power distribution scaling methods on three different types of time–frequency representations (GTX, CTW, and STX). A uniform distribution is used as the global reference distribution, although the methods are transportable to any other distribution. The expected value of information – entropy - is traditionally provided as total quantity over an integration time and/or bandwidth. Traditional entropy studies would have provided a single number for the duration of a signature of interest, or per band or time step. In contrast, the spectral information and entropy SNR quantify these measures at the same resolution as the original time–frequency representation, permitting high-resolution exploration of signature features otherwise occluded by the integration (averaging) process. As discussed at the closing of Section 2, the new instantaneous spectral information SNRs return remarkably stable metrics scaled by the degrees of freedom of the distribution.

Section 3 introduces the Tonga Lamb wave as an exceptionally powerful signal representative of an energetic broadband transient. The New Caledonia pressure record was used because it was well developed in a study by Vergoz et al. [47] and is likely to become a reference signature in future studies. Through a combination of standardized, transportable, and reproducible linear and nonlinear operations, the geophysical pressure signature is transformed into three different instantaneous information and entropy distributions to highlight different aspects of the signal and its ambient noise. The transformation to information unveils otherwise hidden spectral features, thereby increasing the number of choices available for exploratory signal analysis and pattern recognition.

An increase in the level of detail of the relative information and entropy in the signal can be observed in the progression from the Gabor transform, the continuous wavelet transform, and the Stockwell transform, as well the transition from the grid, frequency, and time scaled distribution. One could argue that some of the representations provide too much information; if a sparse representation is desired with only the dominant terms of the main signal, the grid entropy of the CWT may provide the right compromise of energy concentration with minimal time–frequency uncertainty. Only the most stable representations are developed as shown; alternate transformations could be further explored to standardize SNR information and entropy scaling and to provide further enhancements.

As a comparative example, the entropy loss is evaluated for the signal transmission from Tonga to New Caledonia. A geophysical study would treat this as a propagation, problem and would use different metrics and methods. From the entropy perspective, the total grid power distribution reveals when, where, and how much Shannon information is transferred in frequency and time (Figure 13). Although the two recording stations use different types of pressure sensors, their information content can be readily compared. In terms of total entropy, there has been no substantial loss of information; the signals at both stations have ~16 bits of Shannon entropy, and could theoretically be sparsely represented by ~6 Gabor atoms (3 bits/atom). The time of flight, as well as the comparable distribution of cyberspectral information, strongly suggest the two signals are representative of the same source process. Furthermore, since the methods and metrics are well constrained, one may clearly quantify how much information is lost and transformed along the transmission path. A sparse representation of the selected metrics in terms of the dominant quantized Gabor atoms would inherit the minimum time–frequency uncertainty of the atoms, hereby providing standardized, minimal uncertainty estimates of the metrics.

The new scaled spectral information metrics developed in this paper should facilitate more detailed studies on the transformation of signal information along transmission lines. For example, the primary Lamb wave travelled multiple times around Earth. Although theoretically non-dispersive, Lamb wave dispersion and attenuation were observed by hundreds of recording stations during its circumglobal transits. Future exploratory studies could quantify the dissipation and transformation of both primary and secondary Lamb waves into propagating internal, acoustic-gravity and infrasonic waves. The entropy per time step SNR ($esnr_{T}$) may be of particular value for ambient noise studies, whereas the entropy per frequency band SNR ($esnr_{F}$) appears to help enhance high frequencies and may be useful for studies of information transfer into the acoustic regime. The application of the cyberspectral information metrics to less energetic broadband transients, such as those produced by explosives and sonic booms, could be useful for characterizing transmission lines at smaller scales.

These spectral representations can lead to signal-specific methods for feature selection, design, construction, and extraction. The quantized multiresolution framework is versatile and transportable; it has been useful for instrument response comparisons [50] and has been implemented in a smartphone app [51]. How the information in spectral cyberspace is selected and sieved through the filters of our expectations depends on the desired result or intent. The methods presented herein provide enhanced metrics for pattern recognition and feature construction that can be extended to array processing e.g., [36] and used in ML applications.

These methods provide more freedom of choice to select information, a fundamental tenet in signal communication. Signals are considered encoded messages containing information about a source. The propagation of that signal to a digital cyber-physical measurement system is treated as the communication of that information through a transmission channel. In the words of Shannon ([22], p.13), “that information is measured by entropy is, after all, natural when we remember that information, in communication theory, is associated with the amount of freedom of choice we have in constructing messages.”

## Data Availability

The infrasound data used in this study are available as pickled Panda data frames at: https://www.higp.hawaii.edu/archive/isla/Tonga_220115/LAMB_ENTROPY_2023/. These curated data sets include selected instrument-corrected IMS infrasound stations as described in [47], as well as selected stations in the Australian and global barometer networks.

## Appendix A. The Scale Multiplier for the Gabor Atom

As an extension of the Inferno framework [1], the minimal duration (e.g., support) of Gabor atoms can be readily estimated [2] when using standardized fractional octave bands. For a given reference scale $\tau_{0}$, which could be time, frequency, spatial length, wavenumber, or any other useful metric, and a logarithmic scale base *G > 1*, a center time scale $\tau_{n}$ and frequency $\tilde{f}$ can be defined as

(A1) $$\frac{\tau_{n}}{\tau_{0}}=G^{\frac{n}{N}},{\tilde{f}}_{n}=\frac{1}{\tau_{n}}$$

where *n* is the band number and *N* is the band order, subject to the constraints $n\in \mathbb{Z},\;N\geq 1/\sqrt{2}$.

The atom band edges are precisely defined. In the frequency domain, the resulting filter banks overlap close to the −3 dB points, reducing spectral oversampling or leakage. Their scaled bandwidth and quality factor ($Q_{N}$) depends only on the band order

(A2) $$\frac{\Delta \omega_{n}}{\omega_{n}}=\frac{\Delta {\tilde{f}}_{n}}{\;{\tilde{f}}_{n}}=\frac{\;\Delta \tau_{n}}{\;\tau_{n}}=\frac{1}{Q_{N}}\;,\;$$

(A3) $$Q_{N}={\left[\;G^{\frac{1}{2N}}\;–\;G^{-\frac{1}{2N}}\right]}^{-1}\;,\;$$

(A4) $$G=2\;\mathrm{or}\;10^{\frac{3}{10}}\;\mathrm{recommended.}$$

The nominal duration of a constant-Q Gabor atom window $T_{n}$ may be defined as a multiple $M_{N}\;$of the scale as

(A5) $$T_{n}\left(N,n\right)\overset{\mathrm{def}}{=}M_{N}\tau_{n}=M_{N}\tau_{0}G^{\frac{n}{N}}\;$$

where the scale multiplier $M_{N}$ is set by the half power points of the wavelet spectrum. Traditional constant-Q frameworks in acoustics and music applications match the 12-tone equal temperament system (N = 12) for G=2 or $G=10^{\frac{3}{10}}\approx 2$ and are consistent with the Renard series recommended in ISO3 for N = 1, 3, 6, 12, 24. The quality factor $\;Q_{N}$ is the number of oscillations in a little less than half of the total window $T_{n}$, with an amplitude above 1/e of the maximum amplitude. The remaining half of the window is useful to allow the wavelet to settle down and meet the desirable condition of a vanishing first moment. Direct integration of the wavelet power over the window $T_{n}$ shows that it contains 99.999% of all the power [2]. Prior work demonstrated that

(A6) $$Q_{N}=\frac{{\tilde{f}}_{n}}{\Delta {\tilde{f}}_{n}}\approx N\left[\frac{\sqrt{G}}{G-1}\right]\approx \sqrt{2}N\;$$

(A7) $$M_{N}=2\sqrt{ln2}\;Q_{N}\approx 2\sqrt{2ln2}\;N\approx \;2.355\;N\;.$$

This is not an easy relation to remember. An alternate and more memorable approximation (with an error of ~0.05%) is

(A8) $$M_{N}\approx \;\frac{3\pi }{4}\;N=0.75\;\pi N\;\approx 2.356\;N$$

where $M_{N}$ is interpreted as the number of oscillations that will provide adequate support for a Gabor atom. The Gabor–Morlet wavelet scale, which is also the standard deviation of the Gaussian envelope, can be simply expressed in terms of the band order and scale frequency or period as

(A9) $$\sigma_{n}=\frac{M_{N}}{\omega_{n}}=\frac{3\pi }{4}\;\frac{N}{\omega_{n}}=\frac{3}{8}\;\frac{N}{{\tilde{f}}_{n}}=\frac{3}{8}N\;\tau_{n}$$

with $M_{N}$ constant for a specified order N. With this definition, there is no ambiguity on the wavelet minimal support or the number of oscillations in the window. Alternatively, the wavelet support can be expressed in terms of the Gaussian’s standard deviation, which is also the wavelet scale

(A10) $$T_{n}=M_{N}\tau_{n}=2\pi \sigma_{n}\;.$$

From the Gabor uncertainty relation [2], where $\sigma_{t_{n}}$ is the temporal uncertainty

(A11) $$\sigma_{n}=\sqrt{2}\sigma_{t_{n}}$$

(A12) $$T_{n}=2\sqrt{2}\pi \sigma_{t_{n}}\;\sim \;8.886\;\sigma_{t_{n}}\sim \;9\;\sigma_{t_{n}}\;,$$

which is an easy number to remember. In other words, the minimal duration of a signal should be ~9 times its desired temporal uncertainty, regardless of scale or order.

In cyber-physical applications it is often desirable to standardize periods $\tau_{pn}$ and frequencies ${\tilde{f}}_{pn}$ in physical units (e.g., seconds or Hertz, respectively) that are well defined regardless of the sample rate $\;{\tilde{f}}_{s\;}$ in physical units,

(A13) $$\tau_{pn}=\tau_{p0}\;2^{\frac{n}{N}},{\tilde{f}}_{pn}={\tilde{f}}_{p0}\;2^{-\frac{n}{N}}\;,$$

which returns the desired reference or signal frequency at band order *n*=0, with corresponding cyber (nondimensionalized) time scales

(A14) $$\tau_{n}={\tilde{f}}_{s\;}\tau_{pn}=\;{\tilde{f}}_{s\;}\tau_{p0}\;2^{\frac{n}{N}}=\tau_{0}\;2^{\frac{n}{N}}\;.$$

Standardizing to physical frequencies can lead to substantial confusion in the literature and in practical applications. Most mathematical and cyber formulations assume $\;\tau_{0}={\tilde{f}}_{s\;}\tau_{p0}={\tilde{f}}_{s\;}/{\tilde{f}}_{p0}=1$, in which case the frequencies are always relative to Nyquist and the band orders are always positive and greater than the band order *N*. However, if one wants to evaluate at a specific physical period or frequency regardless of the sample rate, additional considerations are needed. For an arbitrary scale base G, we can assert the time scale must be larger than the Nyquist scale

(A15) $$\tau_{n_{min}}>2\;\Rightarrow \;\tau_{0}\;G^{\frac{n_{yq}}{N}}>2$$

(A16) $$n_{min}=n_{nyq}=\mathrm{ceil}\left[\frac{N}{log_{2}\left(G\right)}\left\{1-log_{2}\left(\;{\tilde{f}}_{s\;}/{\tilde{f}}_{p0}\right)\right\}\right]\;.$$

It is pointless to perform multiresolution spectral estimations at or beyond Nyquist. Furthermore, performing computations past the anti-aliasing filters can lead to large roundoff errors that present as artifacts.

As an example, using a nominal anti-aliasing roll off starting at 80% (8/10) of Nyquist

(A17) $$\tau_{n_{min}}>2*\frac{10}{8}\;\Rightarrow \;\tau_{0}\;G^{\frac{n_{aa}}{N}}>2.5$$

(A18) $$n_{min}=n_{aa}=\mathrm{ceil}\left[\frac{N}{log_{2}\left(G\right)}\left\{log_{2}\left(2.5\right)-log_{2}\left(\;{\tilde{f}}_{s\;}/{\tilde{f}}_{p0}\right)\right\}\right]\;.$$

The band number of the standardized frequency closest to any specified frequency is

(A19) $$n=\mathrm{round}\left[\frac{N}{log_{2}\left(G\right)}\left\{log_{2}\left({\tilde{f}}_{pn}/{\tilde{f}}_{p0}\right)\right\}\right]\geq n_{min}\;.$$

The band number of the standardized frequency closest to the physical averaging frequency ${\tilde{f}}_{av}$ is

(A20) $$n_{av}=\mathrm{round}\left[\frac{N}{log_{2}\left(G\right)}\left\{log_{2}\left({\tilde{f}}_{av}/{\tilde{f}}_{p0}\right)\right\}\right]\;,$$

and the recommended dyadic number of points $2^{M}$ needed to support the averaging atom is

(A21) $$log_{2}M=\;\mathrm{ceil}\left[log_{2}\left(M_{N}\tau_{0}\;G^{\frac{n_{av}}{N}}\right)\right]\;.$$

Additionally, the band number corresponding to the largest dyadic number of points closest to the averaging lifetime is

(A22) $$2^{M}\geq \;\max\left[T_{n}\right]=M_{N}\tau_{0}\;G^{\frac{n_{max}}{N}}$$

(A23) $$n_{max}=\mathrm{floor}\left[\frac{N}{log_{2}\left(G\right)}\left\{log_{2}M-log_{2}M_{N}-log_{2}\left({\tilde{f}}_{s\;}/{\tilde{f}}_{p0}\right)\right\}\right]\;.$$

These expressions can be evaluated exactly to obtain the band numbers that define the cyber scales relative to an arbitrary scale base G and physical reference frequency ${\tilde{f}}_{p0}$ sampled at $\;{\tilde{f}}_{s\;}$ and with an averaging frequency ${\tilde{f}}_{av}$. The maximum number of bands will be $n_{max}-n_{min}+1$.

Note that the center frequency and period are a nominal specification that facilitates the computation of the band edge frequencies. However, the Fourier transform is computed in a linear frequency space, and band edge frequencies do not perfectly align when the spectrum peaks at the logarithmic center frequency, which is the geometric mean of the band edges. If one wishes to match the band edges, the specified peak spectrum would be the arithmetic mean of the band edges [1,2]. As discussed in [1], the error is small. However, the center frequency must match the FFT frequencies for amplitude validation; failure to do so will cause instability in amplitude estimates that could be incorrectly attributed to algorithmic or programming errors.

## Appendix B. Fourier Transforms and Convolutions

The symmetric Fourier transform pair

(A24) $${\hat{f}}_{s}\left(\omega \right)=\frac{1}{\sqrt{2\pi }}\int_{-\infty }^{\infty }f\left(t\right)e^{-j\omega t}dt=F_{s}\left\{f\left(t\right)\right\}$$

(A25) $$f\left(t\right)=\frac{1}{\sqrt{2\pi }}\int_{-\infty }^{\infty }{\hat{f}}_{s}\left(\omega \right)e^{j\omega t}d\omega =F_{s}^{-1}\left\{\hat{f}\left(\omega \right)\right\}\;,$$

is favored by mathematicians and leads to a tidy version of the Parseval–Plancherel identity

(A26) $${\left\|{\hat{f}}_{s}\right\|}^{2}={\left\|f\right\|}^{2}\;.$$

The preferred Fourier transform pair used in this work is

(A27) $$\hat{f}\left(\omega \right)=\int_{-\infty }^{\infty }f\left(t\right)e^{-j\omega t}dt=F\left\{f\left(t\right)\right\}$$

(A28) $$f\left(t\right)=\frac{1}{2\pi }\int_{-\infty }^{\infty }\hat{f}\left(\omega \right)e^{j\omega t}d\omega =F^{-1}\left\{\hat{f}\left(\omega \right)\right\}\;,$$

where $\hat{f}\left(\omega \right)$ and $f\left(t\right)$ may be complex, and t and ω are nondimensionalized time and angular frequency, respectively. The variance-scaled time and frequency marginals used to construct information and entropy metrics reduce the importance in the choice of transform as the sum of the marginals will always be unity.

The Discrete Fourier Transform (DFT) pair can be expressed as

(A29) $$\hat{f}\left(\omega_{k}\right)=\sum_{m=0}^{M-1}f\left(m\right)e^{-j\omega_{k}\frac{m}{M}}=F\left\{f\left(m\right)\right\}$$

(A30) $$f\left(m\right)=\frac{1}{M}\sum_{m=0}^{M-1}\hat{f}\left(\omega_{k}\right)e^{j\omega_{k}\frac{m}{M}}=F^{-1}\left\{\hat{f}\left(\omega_{k}\right)\right\}\;,$$

(A31) $$\omega_{k}=2\pi \frac{k}{M},\;k=0,\;1,\;2,\ldots \left(M-1\right)\;.$$

This work uses the Python 3 SciPy [43] implementation of the Fast Fourier Transform (FFT), which matches the response of the FFTW [42]. All further discussions are under the assumption that the number of points *M* is a power of 2. The positive frequencies correspond to the first M/2 points, where we reach the Nyquist frequency. The remaining points correspond to the negative frequency terms. Care should be taken when comparing power and entropy in the time and frequency domain if using the real FFT function, which only returns ½ of the Fourier coefficients, or ½ of the power and entropy.

Useful identities routinely used in Fourier, Wavelet, and S transform analyses are the generalization of the Parseval identity

(A32) $$\int_{-\infty }^{\infty }f\left(t\right)h^{*}\left(t\right)dt=\frac{1}{2\pi }\int_{-\infty }^{\infty }\hat{f}\left(\omega \right){\hat{h}}^{*}\left(\omega \right)d\omega \;,$$

and the related convolution identity

(A33) $$f\left(t\right)*h\left(t\right)=\int_{-\infty }^{\infty }f\left(u\right)\;h^{*}\left(t-u\right)du=\int_{-\infty }^{\infty }f\left(t-u\right)\;h^{*}\left(u\right)du\;.$$

A convolution in the time domain is equivalent to a product in the Fourier domain

(A34) $$F\left\{f\left(t\right)*h\left(t\right)\right\}=F\left\{f\left(t\right)\right\}\;F\left\{h\left(t\right)\right\}\;,$$

so that a convolution can be expressed in terms of the Fourier transform as

(A35) $$f\left(t\right)*h\left(t\right)=F^{-1}\left\{F\left\{f\left(t\right)\right\}\;F\left\{h\left(t\right)\right\}\right\}\;.$$

SciPy [43] provides an efficient FFT convolution algorithm that implements this method with the FFT pairs and is faster than time domain convolution for more than 2^9^ points. Numerical implementation of these transformations requires some care to reduce energy leakage.

## Appendix C. Williams’ Quantification of the Gabor Logon Information

The following expressions are important for quantifying the Gabor logon information and are reproduced here for ready reference. These expressions are based on Williams et al. [9], who follow the nomenclature of Cohen [5].

The instantaneous power per wavelet, or intensity per unit time, is only dependent on the Gaussian envelope

(A36) $$p\left(t\right)={\left|\Psi_{n}\left(t\right)\right|}^{2}={\left|G_{n}\left(t\right)\right|}^{2}=G_{n}^{2}\left(t\right)\;,$$

where the instantaneous power, referred to as the time marginal, is normalized to unity

(A37) $$\int_{-\infty }^{\infty }p\left(t\right)dt=1\;.$$

The symmetric Fourier transform pair (Appendix B)

(A38) $${\hat{G}}_{n}\left(\omega \right)=\frac{1}{\sqrt{2\pi }}\int_{-\infty }^{\infty }G_{n}\left(t\right)\;e^{-j\omega t}dt$$

can be used to obtain the Fourier transform of the Gaussian envelope

(A39) G^nω=σn2π14exp−12ωσn2 .

The spectral energy density, also referred to as the frequency marginal, is

(A40) P^ω=G^nω2=σn2π12exp−ωσn2

where the total energy in the frequency domain is also normalized

(A41) $$\int_{-\infty }^{\infty }\hat{P}\left(\omega \right)d\omega =1\;.$$

The Wigner–Ville [44,45] distribution for one Gabor logon is defined as

(A42) $$W\left(t,\omega \right)=\frac{1}{2\pi }\int_{-\infty }^{\infty }G_{n}\left(t+\tau /2\right)G_{n}^{*}\left(t-\tau /2\right)e^{-i\omega t}d\tau$$

and has a tidy normalized solution

(A43) $$W\left(t,\omega \right)=\frac{1}{\pi }exp\left\{-{\left[\omega \sigma_{n}\right]}^{2}\right\}exp\left\{-{\left[\frac{t}{\sigma_{n}}\right]}^{2}\right\}$$

(A44) $$\iint_{-\infty }^{\infty }W\left(t,\omega \right)d\omega \;dt=1\;,$$

which is a requirement for the use of the time and frequency marginals as probability density functions. Equations (A36)–(A41) are the historically preferred forms for the computation of Gabor entropy [9] for statisticians or mathematicians.

The Shannon information [22,23] $I_{t}$ is expressed in bits and defined as the binary logarithm of the probability p, which can be represented by the marginal

(A45) $$I_{t}=log_{2}\;\left(1/p\right)=-log_{2}\;p\;.$$

The Shannon entropy $H_{t}$ is also in bits and is the expected value ($E_{p}$) over the distribution and is expressed in various familiar forms [22,25],

(A46) $$H_{t}=E_{p}\left(I_{t}\right)$$

(A47) $$H_{t}=\int_{-\infty }^{\infty }I_{t}dp$$

with the familiar discrete representation

(A48) $$H_{t}=-\sum_{i}p_{i}\;log_{2}\;p_{i}\;.$$

This scaling was selected by Shannon [22] so that a binary system, such as the flip of a coin, will have one bit of information and one bit of entropy. Although the terms for entropy and information are often used interchangeably e.g., [24], the distinction is maintained in this paper.

Williams et al. [9] compute the Shannon entropy per Gabor logon using the marginals in the time domain

(A49) $$H_{t}=-\int_{-\infty }^{\infty }p\left(t\right)log_{2}\;p\left(t\right)\;dt\;,$$

(A50a) $$H_{t}=\frac{log_{2}\pi }{2}+\frac{log_{2}e}{2}+log_{2}\sigma_{n}\;,$$

(A50b) $$H_{t}=log_{2}\left[\sqrt{\pi e}\right]+log_{2}\sigma_{n}\;,$$

and in the frequency domain

(A51a) $$H_{\omega }=-\int_{-\infty }^{\infty }\hat{P}\left(\omega \right)log_{2}\;\hat{P}\left(\omega \right)\;d\omega \;,$$

(A51b) $$H_{\omega }=log_{2}\left[\sqrt{\pi e}\right]-log_{2}\sigma_{n}\;,$$

where one may note that, like the uncertainty, the joint information in the time and frequency domains remains constant.

Therefore, the total entropy in the Wigner–Ville time–frequency distribution is

(A52a) $$H_{t\omega }=\iint_{-\infty }^{\infty }W\left(t,\omega \right)\;log_{2}W\left(t,\omega \right)\;d\omega \;dt$$

(A52b) $$H_{t\omega }=log_{2}\pi +log_{2}e=log_{2}\left[\pi e\right]\;\sim \;3\;$$

where the authors [9] conclude that the Wigner distribution provides no additional information about a signal beyond that provided by the marginals

(A53) $$H_{t\omega }-\left(H_{t}+H_{\omega }\right)=0\;.$$

This important result can be extended to the time–frequency uncertainty. For a discrete (e.g., cyber-physical digital) sensor system, the logon information can be estimated from the marginals with

(A54a) $$H_{t}=-\sum_{i}p_{i}\;log_{2}\;p_{i}=\sum_{i}h_{t}\left(i\right)$$

(A54b) $$H_{\omega }=-\sum_{k}{\hat{P}}_{k}\;log_{2}\;{\hat{P}}_{k}=\sum_{k}h_{\omega }\left(k\right)$$

and can be compared to the theoretical continuous time values for consistency scaling,

(A55) $$H_{t}-log_{2}\left[\sqrt{\pi e}\right]=log_{2}\sigma_{n}$$

(A56) $$log_{2}\left[\sqrt{\pi e}\right]-H_{\omega }=log_{2}\sigma_{n}$$

where

(A57) $$log_{2}\left[\sqrt{\pi e}\right]\;\sim \;3/2,\;\;log_{2}\left[\pi e\right]\;\sim \;3\;,$$

Note, the information per logon in the time or frequency domain has a characteristic scale

(A58a) $$log_{2}\sigma_{n}=log_{2}\left(\frac{M_{N}}{\omega_{n}}\right)=log_{2}\left(\frac{3N}{8}\right)-log_{2}\frac{f_{p}}{f_{s}}$$

(A58b) $$=1+log_{2}\left(\frac{3N}{8}\right)-log_{2}\frac{f_{p}}{f_{Nyq}}$$

where $f_{p}<f_{s}/2$ = Nyquist frequency $f_{Nyq}$.
